# Design and Synthesis of Geranylated Benzaldehydes: Evaluation of Antifungal Activities Against *Botrytis cinerea* and *Phytophthora cinnamomi*

**DOI:** 10.3390/molecules31193481

**Published:** 2026-09-29

**Authors:** Ligia Llovera, Luis Espinoza-Catalán, Héctor Carrasco, Andrés F. Olea, Marco Mellado, Lautaro Taborga, Mauricio Soto

**Affiliations:** 1 Departamento de Química, Universidad Técnica Federico Santa María, Av. España No. 1680, Valparaíso 2340000, Chile; ligia.llovera@usm.cl (L.L.); luis.espinozac@usm.cl (L.E.-C.); 2 Grupo QBAB, Instituto de Ciencias Aplicadas, Facultad de Ingeniería, Universidad Autónoma de Chile, Av. del Valle Sur 534, Santiago 8580640, Chile; hector.carrasco@uautonoma.cl (H.C.); andres.olea@uautonoma.cl (A.F.O.); 3 Dirección de Investigación, Universidad Bernardo O’Higgins, Santiago 8370993, Chile; marco.mellado@ucentral.cl; 4 Laboratorio de Espectroscopía y Química Aplicada, Grupo de Investigación en Ciencias Biomédicas Aplicadas, Universidad Central de Chile, Santiago 8330507, Chile; 5 Departamento de Ciencias Básicas, Facultad de Ciencias, Universidad Santo Tomás, Viña del Mar 2561694, Chile

**Keywords:** geranylated benzaldehydes, antifungal activity, *Botrytis cinerea*, *Phytophthora cinnamomi*, succinate dehydrogenase, molecular docking, suzuki cross-coupling, S_N_2 reaction

## Abstract

A series of ten geranylated benzaldehydes (**11**–**20**) was designed, synthesized via Suzuki cross-coupling for the *C*–*C* series and S_N_2 substitution for the *C*–*O* derivatives, and structurally characterized. The antifungal activity of all compounds against the phytopathogens *Botrytis cinerea* and *Phytophthora cinnamomi*, two important plant pathogens associated with substantial agricultural and economic losses worldwide, was evaluated in vitro using a mycelial growth inhibition assay. Dose–response treatment of the data indicates that (E)-2-((3,7-dimethylocta-2,6-dien-1-yl)oxy)-5-hydroxybenzaldehyde (**19**) is the most active compound exhibiting EC_50_ values of 83 µg/mL and 7.34 µg/mL against *B. cinerea* and *P. cinnamomi*, respectively. To gain insight into the molecular basis of the observed antifungal activity, molecular docking studies were performed targeting the active site of succinate dehydrogenase (SDH, PDB ID: 2FBW), a key enzyme in the mitochondrial respiratory chain and a validated fungicide target. The calculated docking scores ranged from −6.0 to −7.5 kcal/mol, with compound **19** showing the highest binding affinity. Docking analysis reveals that compound **19** is stabilized within the SDH binding pocket through a network of hydrogen bonding and π–cation interactions involving the heme prosthetic group and key amino acid residues, providing a plausible structural rationale for its high antifungal activity, particularly against *P. cinnamomi*.

## 1. Introduction

*Botrytis cinerea* and *Phytophthora cinnamomi* are among the most destructive phytopathogens affecting agricultural and forestry production worldwide. *Botrytis cinerea*, the causal agent of gray mold disease, infects more than 200 plant species and causes severe economic losses during cultivation, storage, and postharvest handling [1,2]. Likewise, *P. cinnamomi* is responsible for root rot and dieback diseases in numerous crops and forest species, generating significant economic and ecological impacts [3,4]. The extensive use of conventional fungicides has promoted the emergence of resistant populations, reduced the effectiveness of several commercial products, and created an urgent need for new antifungal agents with alternative chemical structures and mechanisms of action [5,6].

Geranylated aromatic compounds constitute a structurally diverse family of natural products that have been isolated from marine organisms, terrestrial plants, and fungi [7,8,9,10,11,12,13,14,15,16,17,18]. These metabolites have attracted considerable attention because they exhibit a broad range of biological properties, including antimicrobial, antifungal, antiviral, antiparasitic, and anticancer activities [19,20,21,22,23,24,25,26]. Representative examples of naturally occurring *C*-geranylated aromatic compounds with relevant biological activities are shown in Figure 1. The combination of an aromatic nucleus with a lipophilic geranyl side chain provides a versatile scaffold capable of interacting with biological membranes and molecular targets, making these compounds attractive templates for the development of new bioactive molecules.

Several studies have demonstrated that geranylated phenolic derivatives possess significant antifungal activity against phytopathogenic fungi. *C*-geranylated hydroquinone’s, resorcinol’s, and related aromatic compounds have been reported to inhibit the growth of *B. cinerea* and *P. cinnamomi* [27,28,29,30,31,32]. Structure–activity relationship studies suggest that the antifungal properties of these compounds are strongly influenced by the substitution pattern of the aromatic ring, the presence of hydroxyl groups, and the bonding nature by which the geranyl moiety is attached to the aromatic scaffold. Selected examples of synthetic biologically active prenylated aromatic compounds reported in the literature are presented in Figure 2.

Beyond their direct antifungal activity, several geranylated aromatic metabolites have recently been identified as inhibitors of succinate dehydrogenase (SDH), an essential enzyme involved in both the tricarboxylic acid cycle and fungal mitochondrial respiration [33,34]. SDH inhibitors (SDHIs) constitute one of the most successful classes of modern fungicides due to their broad-spectrum activity against economically important phytopathogens, including *Botrytis cinerea* and *Phytophthora* species. In addition to that SDH is a validated target for several commercial fungicides, structurally related prenylated and geranylated aromatic compounds have shown important antifungal activities against both fungi [28,35]. These findings suggest that geranylated aromatic compounds might serve as promising lead structures for the development of novel antifungal agents.

In this context, and in the absence of direct biochemical evidence identifying the molecular target of geranylated benzaldehydes, succinate dehydrogenase (SDH) was selected as a target for molecular docking study. Molecular docking has become a valuable tool for exploring ligand–protein interactions and generating hypotheses regarding the molecular basis of biological activity [36,37,38].

Moreover, recent studies have suggested that lipophilic aromatic compounds bearing hydrogen-bond donor and acceptor groups can interact with the ubiquinone-binding region of SDH, providing a structural basis for evaluating related scaffolds through computational approaches [39]. Therefore, SDH appears to be a suitable biological target to explore possible ligand–protein interactions and generate mechanistic hypotheses regarding the antifungal activity of the synthesized compounds. It should be emphasized that the docking analysis presented herein is intended to provide structural insight into potential binding modes rather than experimental proof of the molecular mechanism, which will require further enzymatic and biochemical validation.

Thus, a series of novel geranylated benzaldehydes, **11**–**20**, with different substitution patterns and modes of geranyl attachment, i.e., either carbon–carbon or carbon–oxygen bonds, have been designed and synthesized. The latter structural feature has not been considered in previous work, and we think that it is worth to assess the effect of geranyl linkage on antifungal activity and, in this way, to gain preliminary insights on their contribution to structure–activity relationship (SAR).

The antifungal activity of these compounds against *B. cinerea* and *P. cinnamomi* was evaluated by using mycelial growth inhibition assays. In addition, molecular docking studies were performed using succinate dehydrogenase as a potential molecular target to explore possible structure–activity relationships and provide insights into the observed antifungal activity.

## 2. Results and Discussion

### 2.1. Synthesis

All synthesized compounds (**11**–**20**) are novel (see Figure 3), and the synthetic procedure depends mainly on the kind of bonding of the geranyl moiety to the aromatic ring. A variety of synthetic strategies have been reported to obtain geranylated aromatic compounds, involving either carbon–carbon (*C*–*C*) or carbon–oxygen (*C*–*O*) bond formation. *C*–*C* coupled geranylated aromatics are commonly obtained via Friedel–Crafts alkylation, transition-metal-catalyzed cross-coupling reactions, or electrophilic aromatic substitution using geranyl halides or related electrophiles [40,41,42,43,44,45,46]. Although, *C*–*O* coupled derivatives are typically prepared through *O*-alkylation of phenolic substrates with geranyl halides under basic conditions, providing access to geranyl ethers [47,48]. These complementary synthetic approaches have enabled the efficient preparation of structurally diverse geranylated aromatic frameworks and facilitated systematic studies of their biological activities.

Herein, *C*-geranylated derivatives (**11**–**13**) were obtained by reacting substituted arylboronic acids with geranyl bromide through a Suzuki coupling reaction in the presence of a palladium catalyst. The synthetic route for the preparation of compound **12** is shown in Scheme 1, as an example.

The low yields observed for isolated compounds **11**–**13** (18–26%) are consistent with the inherent challenges of using allylic bromides as electrophilic partners in palladium-catalyzed cross-coupling reactions. Unlike aryl or vinyl halides, allylic substrates such as geranyl bromide undergo oxidative addition to Pd(0) to generate a η^3^-π-allylpalladium(II) intermediate. In this species, the electrophilic character is delocalized across both termini of the allyl system (the α and γ carbons) [49]. This delocalization allows the nucleophile to attack either substrate’s ends, resulting in a mixture of regioisomeric coupling products. This is evidenced by the formation of isomers **12** and **22**, as depicted in Scheme 1, when (4-formyl-2-methoxyphenyl)boronic acid is used as the starting material.

The formation of 2-methoxybenzaldehyde (**23**) as a byproduct via the protodeboronation of boronic acid **21** is a well-documented side reaction in Suzuki–Miyaura couplings [50]. Protodeboronation of arylboronic acids can proceed through multiple pathways, including uncatalyzed reaction with water, as well as acid-, base-, and metal-catalyzed mechanisms; the base-catalyzed pathway is particularly relevant under standard cross-coupling conditions [51]. Electron-rich arylboronic acids, especially those bearing methoxy substituents, are highly susceptible to this competitive pathway, which directly opposes productive transmetalation and contributes to the diminished yields of the target coupling product [50,52]. Thus, the susceptibility of **21** to protodeboronation is consistent with its electronic profile and represents a primary factor limiting the efficiency of this transformation.

Compounds **12** and **22** are regio- and geometric isomers differing only in the position or configuration of a double bond within a lipophilic terpene chain. Therefore, their polarity is nearly identical and consequently, their separation from the product mixture by standard silica gel chromatography was not possible. Thus, silver nitrate-impregnated silica gel, which leverages the reversible coordination of Ag^+^ ions with π-electron systems to differentiate isomers based on the stability of their Ag^+^–olefin complexes, has been used to achieve partial isolation of **12** from **22** [53,54]. Thus, these spectroscopically pure fractions were collected, pooled, and subsequently used in the biological assays. This agrees with previous reports showing that the resolution of conformationally flexible acyclic polyenes usually demands precise optimization of silver impregnation levels, solvent gradients, or column dimensions [55].

On the other hand, *O*-geranylated benzaldehydes (**14**–**20**) were synthesized via bimolecular nucleophilic substitution (S_N_2) reaction between various substituted benzaldehydes and geranyl bromide, in the presence of K_2_CO_3_ and using DMF as the solvent, affording isolated yields of 45–95%. The synthetic route for the preparation of compound **15** is shown in Scheme 2, as an example.

This transformation follows a Williamson ether synthesis protocol, in which the phenolic hydroxyl group of the starting material acts as the nucleophile upon in situ deprotonation by K_2_CO_3_ to generate the corresponding phenoxide ion. Although K_2_CO_3_ is a relatively mild base (pKa 10.3), it is sufficient to deprotonate these phenolic substrates. For example, 4-hydroxy-3,5-dimethoxybenzaldehyde (**24**) was reacted with geranyl bromide in the presence of K_2_CO_3_ in DMF at 130 °C, providing **15** in 87% yield after purification by column chromatography, Scheme 2. In this reaction, geranyl bromide acts as an allylic electrophile; the bromide leaving group is positioned at the primary allylic carbon (α-position), making it highly susceptible to S_N_2 attack. The primary nature of this α -carbon facilitates direct displacement, consistent with the high yield observed. Heating at elevated temperature (130 °C) in DMF, characteristic of Williamson syntheses using mild inorganic bases, is required to compensate for the equilibrium-limited generation of phenoxide and to overcome the activation energy of the substitution step.

The chemical structures of the synthesized geranylated benzaldehydes were elucidated primarily through comprehensive nuclear magnetic resonance (NMR) analyses, including ^1^H, ^13^C, and two-dimensional (2D) experiments. The combined interpretation of ^1^H and ^13^C NMR spectra with 2D techniques such as HSQC and HMBC enabled unambiguous assignment of proton–carbon correlations and allowed the definitive structural characterization of the compounds. The connectivity between the aromatic ring and the geranyl moiety is primarily evidenced by the chemical shift in the H-1′ methylene protons. In C–C coupled geranylated benzaldehydes, this signal appears at approximately 3.30 ppm, consistent with an aliphatic methylene adjacent to an sp^2^ carbon. In contrast, for C–O coupled derivatives, the H-1′ resonance shifts downfield to around 4.50 ppm, reflecting the increased deshielding effect associated with the oxygen substituent. This effect on chemical shift is exemplified by comparing NMR spectra of compounds **19** and **11** (Figure 4). For **19**, ether formation was confirmed by a characteristic benzylic O–CH_2_ signal at δ_H_ 4.6 ppm with its corresponding carbon resonance at δ_C_ 66.3 ppm in the HSQC spectrum, while for **11**, C–C bond formation was demonstrated by a benzylic methylene signal (CH_2_–Ar) at δ_H_ 3.42 ppm (δ_C_ 25.7 ppm).

### 2.2. In Vitro Antifungal Activity

The antifungal activity of all synthesized geranylated benzaldehyde derivatives (**11**–**20**) was evaluated against *B. cinerea* and *P. cinnamomic*, by measuring mycelial growth inhibition at different concentrations of tested compounds. The results are given in Table 1 and Table 2 as inhibition percentage for *B. cinerea* and *P. cinnamomic*, respectively.

The data obtained for *B. cinerea*, show that compounds **11**–**20** displayed a wide range of inhibitory activities, with growth inhibition values ranging from 0 to 74% over the tested concentration range (10–160 µg/mL). A careful analysis of the data in Table 1 reveals a clear dependence of activity with the structural features of each compound. Apparently, the substitution pattern on the aromatic ring, the nature of the geranyl attachment (*C*-*C* vs. *C*-*O*), and the presence or absence of a free phenolic hydroxyl group are important parameters to be considered.

Compounds **11**–**13** possess aromatic ring substituted with a methoxy group and the geranyl chain directly attached by *C*-*C* bonding. Their inhibitory activities are moderate and essentially concentration-independent, with similar maximum values at 160 µg/mL, i.e., 18.1%, 23.0%, and 26.2%, respectively. On the other hand, compounds **15**–**19** are O-geranylated derivatives, and **15** exhibited the highest activity at all tested concentrations. A structural comparison between **15** and **16** show that the only difference is an extra methoxy group in **15** (Figure 3).

The highest activity of compound **15** may be attributed to the synergistic electronic effects of its two methoxy groups at the 3- and 4-positions of the aromatic ring. In related geranylated phenol series evaluated against *B. cinerea*, antifungal activity has been reported to increase progressively with the number of methoxy groups on the aromatic ring, rising from 40% to 90% inhibition as substitution increases [28]. The two methoxy groups in **15** may increase the electron density of the aromatic ring through resonance effects, which could favor interactions with biological targets. Thus, the weak activity of **14**, **18**, and **20** can be primarily attributed to the absence of electron-donating substituents on the aromatic ring. In these compounds, the only ring substituents are the electron-withdrawing formyl group and the *O*-geranyl chain. The electron density of the aromatic ring is significantly diminished by the inductive and mesomeric electron-withdrawal of the aldehyde moiety. This electronic depletion likely reduces the capacity of the molecules to interact productively with their biological targets or active-site residues.

Finally, compounds **17** and **19** display the same functional groups, although they are located at different positions on the aromatic ring. The antifungal activity of compound **19** at 160 µg/mL (62.2% inhibition; Figure S58), relative to its regioisomer **17** (16.8% inhibition) highlights that the positional availability of the free hydroxyl group is as decisive as its presence. In compound **17**, the ortho-OH/CHO arrangement gives rise to a strong intramolecular hydrogen bond that sequesters the phenolic -OH group, restricting its ability to interact with external biological targets. Conversely, the -OH group in compound 19 is geometrically remote from the carbonyl moiety, preventing intramolecular chelation. This leaves the -OH fully exposed to participate in intermolecular hydrogen bonding with the active site, while the ortho-geranyloxy chain engages in hydrophobic interactions. This mode of action accounts for the superior potency and enhanced pharmacophoric fit of compound **19** relative to **17**.

In conclusion, while the digeranylated compound **20** is completely inactive at all tested concentrations, compounds **15** and **19** are the most active derivatives in the entire series, achieving growth inhibitions of 74.0% and 62.2% at 160 μg/mL, respectively, and demonstrating a clear concentration-dependent profile.

The data of antifungal activity of compounds **11**–**20** against *Phytophthora cinnamomi* is summarized in Table 2 and reveal a broad spectrum of inhibitory potency among the tested derivatives, with compound **19** exhibiting the most pronounced activity, followed by compounds **14** and **17**, across all tested concentrations. These findings highlight the critical influence of substituent pattern on the benzaldehyde scaffold in determining biological efficacy.

Compounds **11**–**13**, which feature a C–C linked geranyl side chain alongside methoxy and formyl substituents directly on the aromatic ring, displayed uniformly low inhibitory activity, with maximum values of 5.2%, 3.7%, and 3.6% at 50 µg/mL for compounds **12**, **11**, and **13**, respectively. On the other hand, *O*-geranylated derivatives (**14**–**18** and **20**), compound **14**) exhibited a strictly concentration-dependent response, increasing from 2.4% inhibition at 3 µg/mL to 34.7% at 50 µg/mL. Compound **17** showed negligible activity at lower concentrations (0% at 3 and 6 µg/mL) but recovered substantially at higher doses, reaching 44.7% inhibition at 50 µg/mL. This behavior may suggest a concentration threshold effect, likely influenced by the intramolecular hydrogen bonding between the -OH and the formyl group. Compounds **16** and **15**, which bear one and two methoxy groups, respectively, in addition to the geranyloxy chain, displayed moderate activity with maximum inhibitions of 10.0% and 23.8% at 50 µg/mL. Finally, compound **20**, a bis-etherified derivative carrying two geranyloxy chains, exhibited low activity within the etherified series (15.1% across all concentrations).

Compound **19**, an *O*-geranylated benzaldehyde derivative bearing a free hydroxyl group, was the most active of the series, displaying potent, concentration-dependent inhibition against *Phytophthora cinnamomi* growth.

Interestingly, at the lowest concentration tested (3 µg/mL), compound **19** achieved 34.4% inhibition, a level not reached by any other derivative even at 50 µg/mL. Inhibition increased sharply to 56.8% at 6 µg/mL and exceeded 86% at 12 µg/mL, approaching a plateau at higher concentrations (89.3% and 88.3% at 25 and 50 µg/mL, respectively; Figure S59), with an EC_50_ value of 7.34 µg/mL confirming its high intrinsic potency. These results are consistent with previous reports indicating that geranylated derivatives bearing hydroxyl groups attached to the aromatic ring, such as compound **5**, constitute the most efficient inhibitors within this class of compounds [32], as evidenced by their inhibition levels against *P. cinnamomi* (86% at 50 µg/mL). This effect is primarily attributed to changes in the electronic distribution of the aromatic ring induced by the nature and position of the substituents.

Notably, the EC_50_ value obtained for compound **19** is within the same concentration range as those reported for several commercial fungicides (Table 3), despite its considerably simpler molecular architecture and the absence of any prior structural optimization. Although the compound does not yet match the potency of the most active SDHI fungicides, its biological performance is sufficiently encouraging to identify this geranylated benzaldehyde as a promising lead structure for further optimization. Small structural modifications around the aromatic substitution pattern and the geranyl moiety may therefore provide an effective strategy for improving antifungal potency while preserving the favorable activity profile observed in the present study.

Specifically, the high potency of compound **19** correlates closely with the results found for well-established *C*-geranylated resorcinol, such as compounds **25** and its methylated analogue **5**, which have demonstrated remarkable efficacy in the literature. The fact that compound **19** elicits such strong inhibition highlights that the presence of the geranyl chain remains a critical structural requirement for activity, regardless of whether it is attached via a direct C-C bond or an ether link (*O*-alkylation). Furthermore, unlike traditional resorcinol scaffolds, compound **19** features a strategic formyl group (-CHO) adjacent to the geranyloxy chain. This electron-withdrawing substituent significantly alters the electrostatic surface potential of the ring, potentially modulating the acidity of the remaining hydroxyl group while acting as a strong hydrogen-bond acceptor that can facilitate crucial interactions within the target binding site. Consequently, the optimal substitution pattern of compound **19**, which combines a geranyloxy chain with a free hydroxyl group, may contribute to its superior performance relative to the other derivatives in the series, as further highlighted by the markedly lower activity of its regioisomer, compound **17**.

Overall, the results reveal a clear species-dependent antifungal response, with compound **19** showing markedly higher potency against *P. cinnamomi* (EC_50_ = 7.34 µg/mL) than against *B. cinerea* (EC_50_ = 83.0 µg/mL). This difference may be partly related to the distinct cell-wall architecture and membrane properties of these phytopathogens, which could affect compound uptake and accessibility to cellular targets [60,61]. However, as these parameters were not directly investigated in the present study, this interpretation should be considered a plausible explanation rather than a demonstrated mechanism.

### 2.3. Molecular Docking Study

Given the significant and species-dependent antifungal activity observed for the synthesized geranylated benzaldehydes, molecular docking studies were performed to investigate a plausible molecular basis for their biological activity. Since the molecular target of these compounds has not yet been experimentally established, succinate dehydrogenase (SDH) was selected as a biologically relevant target because it is the molecular target of commercially important SDHI fungicides and has previously been proposed as a potential target for structurally related prenylated aromatic compounds. Accordingly, the docking analysis should be interpreted as a hypothesis-generating approach aimed at identifying potential ligand–protein interactions that may contribute to the observed antifungal activity, rather than as direct evidence of the mechanism of action. The calculated docking scores ranged from −6.0 to −7.5 kcal/mol (Table S2, Figures S60–S69), with compound **19** exhibiting the most favorable binding affinity (−7.5 kcal/mol). This result is consistent with its high inhibition percentage in the screening assays and its low EC_50_ value against *P. cinnamomi* and *B. cinerea*, suggesting a favorable interaction with the SDH binding pocket.

Inspection of the docking pose of compound **19** showed that its functional groups were appropriately oriented within the SDH binding pocket to establish several potential stabilizing interactions (Figure 5). The docking model predicts that the carbonyl group may participate in two hydrogen-bond interactions, while the geranyl chain is predicted to occupy a neighboring hydrophobic pocket, potentially contributing to ligand stabilization. In addition, the hydroxyl group at position 5 is predicted to establish a hydrogen bond with the heme (HEM) prosthetic group, whereas the aromatic ring may engage in a π–cation interaction with Arg43. Collectively, these predicted interactions could contribute to the favorable binding pose and may help to explain the superior antifungal activity observed for compound **19**. Nevertheless, these computational results should be regarded as a hypothesis-generating model, and experimental validation through biochemical or enzymatic assays will be necessary to confirm the proposed binding mode and molecular mechanism of action.

## 3. Materials and Methods

### 3.1. General

All reagents were purchased from Merck (Darmstadt, Germany) or Sigma-Aldrich (St. Louis, MO, USA) and used as received, without additional purification. ^1^H, ^13^C, 2D HSQC and 2D HMBC NMR spectra were acquired on a Bruker Avance 400 spectrometer operating at 400.1 MHz for ^1^H and 100.6 MHz for ^13^C. Samples were dissolved in CDCl_3_, and chemical shifts (δ) are reported in ppm; coupling constants (J) are expressed in Hz. Chemical shifts were referenced to the residual CHCl_3_ signal at δ = 7.26 ppm ^1^H) and δ = 77.00 ppm ^13^C. High-resolution mass spectra (HRMS-ESI) were recorded in a Bruker Daltonik (Bruker, Bremen, Germany). Pure products were isolated via column chromatography on silica gel (Merck, 200–300 mesh). Reaction progress was monitored by thin-layer chromatography (TLC) on silica gel plates (HF-254), and spots were visualized by heating after spraying with 25% H_2_SO_4_.

### 3.2. Synthesis

#### 3.2.1. General Procedure for C-Geranylation of Benzaldehydes

Compounds **11**–**13** were synthesized via a Suzuki–Miyaura cross-coupling reaction between the corresponding arylboronic acids and geranyl bromide. The appropriate arylboronic acid (1.0 equiv), geranyl bromide (1.2–1.5 equiv), and a base (2.0–3.0 equiv) were dissolved or suspended in a degassed mixture of anhydrous solvent under an inert atmosphere. A palladium catalyst was then added, and the reaction mixture was heated under reflux with stirring for the required reaction time, as monitored by thin-layer chromatography (TLC).

After completion, the reaction mixture was cooled to room temperature, diluted with water, and extracted with ethyl acetate. The combined organic layers were washed with brine, dried over anhydrous MgSO_4_, filtered, and concentrated under reduced pressure. The crude products were initially purified by silica gel 60 column chromatography; however, in all cases, the coupling afforded mixtures of regioisomeric products that could not be completely resolved under standard conditions. Partial separation of the coupled isomers was achieved using silica gel 60 impregnated with silver nitrate (AgNO_3_), which allowed the isolation of some purified fractions.

#### 3.2.2. General Procedure for O-Geranylation of Benzaldehydes

A mixture of the corresponding hydroxy-substituted benzaldehyde precursor (1.0 equiv) and potassium carbonate (2.0–3.0 equiv) was suspended in *N*,*N*-dimethylformamide (DMF). The reaction mixture was stirred at room temperature for 10–15 min, after which geranyl bromide (1.1–1.3 equiv) was added dropwise. The resulting mixture was heated at 130 °C and stirred for the appropriate reaction time, as monitored by thin-layer chromatography (TLC). Upon completion, the reaction was allowed to cool to room temperature and quenched with distilled water. The aqueous phase was extracted with ethyl acetate, and the combined organic layers were washed with water and brine, dried over anhydrous MgSO_4_, filtered, and concentrated under reduced pressure. The crude product was purified by silica gel column chromatography using a mixture of n-hexane/ethyl acetate as eluent to afford the corresponding geranylated benzaldehydes **14**–**20** in moderate to excellent yields (45–95%).

*(E)-3-(3,7-Dimethylocta-2,6-dien-1-yl)-2-methoxybenzaldehyde* (**11**)

Compound **11** was obtained from (3-formyl-2-methoxyphenyl)boronic acid (1.0 g, 5.5 mmol) and geranyl bromide (1.4 g, 6.6 mmol) in acetone (20 mL). Compound **11** was isolated as a pale-yellow oil (317 mg, 21% yield). ^1^H NMR (400 MHz, CDCl_3_): δ 10.38 (s, 1H, –CHO); 7.48 (dd, *J* = 7.7 and 1.5 Hz, 1H, H-6); 7.46 (d, *J* = 7.5 Hz, 1H, H-4); 7.17 (t, *J* = 7.6 Hz, 1H, H-5); 5.28 (m, 1H, H-2′); 5.09 (m, 1H, H-6′); 3.90 (s, 3H, CH_3_O-C2); 3.42 (d, *J* = 7.0 Hz, 2H, H-1′); 2.12–2.04 (m, 4H, H-5′ and H-6′); 1.73 (s, 3H, CH_3_-C3′); 1.67 (s, 3H, H-8′); 1.59 (s, 3H, CH_3_-C7′).^13^C NMR (100 MHz, CDCl_3_): δ 190.3 (C=O); 161.4 (C-2); 137.2 (C-3); 136.5 (C-4); 136.1 (C-3′); 131.6 (C-7′); 129.2 (C-1); 126.8 (C-6); 124.6 (C-5); 124.1 (C-6′); 121.9 (C-2′); 64.0 (OCH_3_); 39.7 (C-4′); 27.4 (C-5′); 26.5 (C-8′); 25.7 (C-1′); 17.7 (CH_3_-C7′); 16.2 (CH_3_-C3′). HRMS (ESI^+^): *m*/*z* calcd. for C_18_H_24_O_2_ [M + H]^+^: 273.1849; found: 273.1845. (see Figure S48, Supplementary Material).

*(E)-4-(3,7-dimethylocta-2,6-dien-1-yl)-3-methoxybenzaldehyde* (**12**)

Compound **12** was obtained from (4-formyl-2-methoxyphenyl)boronic acid (1.0 g, 5.5 mmol) and geranyl bromide (1.5 g, 6.9 mmol) in acetone (20 mL). Compound **12** was isolated as a pale-yellow oil (396 mg, 26% yield). ^1^H NMR (400 MHz, CDCl_3_): δ 9.93 (s, 1H, –CHO); 7.39 (dd, *J* = 7.6 and 1.4 Hz, 1H, H-6); 7.35 (d, *J* = 1.4 Hz, 1H, H-2); 7.30 (d, *J* = 7.6 Hz, 1H, H-5); 5.30 (m, 1H, H-2′); 5.10 (m, 1H, H-6′); 3.9 (s, 3H, CH_3_O-C3); 3.38 (d, *J* = 7.3 Hz, 2H, H-1′); 2.12–2.08 (m, 4H, H-5′ and H-4′); 1.68 (s, 6H, CH_3_-C3′ and H-8′); 1.60 (s, 3H, CH_3_-C7′).^13^C NMR (100 MHz, CDCl_3_): δ 190.3 (C=O); 157.9 (C-3); 138.1 (C-1); 137.4 (C-4); 135.7 (C-3′); 131.5 (C-7′); 129.2 (C-5); 124.6 (C-6); 124.2 (C-6′); 120.9 (C-2′); 108.1 (C-2); 55.5 (OCH_3_); 39.7 (C-4′); 28.6 (C-1′); 26.6 (C-5′); 25.7 (C-8′); 17.7 (CH_3_-C7′); 16.1 (CH_3_-C3′). HRMS (ESI^+^): *m*/*z* calcd. for C_18_H_24_O_2_ [M + H]^+^: 273.1849; found: 273.1847. (see Figure S49, Supplementary Material).

*(E)-5-(3,7-dimethylocta-2,6-dien-1-yl)-2-methoxybenzaldehyde* (**13**)

Compound **13** was obtained from (3-formyl-4-methoxyphenyl)boronic acid (1.0 g, 5.5 mmol) and geranyl bromide (1.6 g, 7.4 mmol) in acetone (20 mL). Compound **13** was isolated as a pale-yellow oil (272 mg, 18% yield). ^1^H NMR (400 MHz, CDCl_3_): δ 10.44 (s, 1H, –CHO); 7.63 (d, *J* = 2.3 Hz, 1H, H-6); 7.36 (dd, *J* = 8.6 and 2.3 Hz, 1H, H-4); 6.90 (d, *J* = 8.6 Hz, 1H, H-3); 5.28 (m, 1H, H-2′); 5.09 (m, 1H, H-6′); 3.91 (s, 3H, CH_3_O-C2); 3.32 (d, *J* = 7.3 Hz, 2H, H-1′); 2.11–2.04 (m, 4H, H-5′ and H-4′); 1.70 (s, 3H, CH_3_-C3′); 1.67 (s, 3H, H-8′); 1.59 (s, 3H, CH_3_-C7′). ^13^C NMR (100 MHz, CDCl_3_): δ 190.1 (C=O); 160.2 (C-2); 136.7 (C-3′); 135.9 (C-4); 134.2 (C-1); 131.6 (C-7′); 128.0 (C-6); 124.6 (C-5); 124.2 (C-6′); 122.6 (C-2′); 111.7 (C-3); 55.7 (OCH_3_); 39.7 (C-4′); 33.1 (C-5′); 26.6 (C-8′); 25.7 (C-1′); 17.7 (CH_3_-C7′); 16.2 (CH_3_-C3′). HRMS (ESI^+^): *m*/*z* calcd. for C_18_H_24_O_2_ [M + H]^+^: 273.1849; found: 273.1848. (see Figure S50, Supplementary Material).

*(E)-4-((3,7-dimethylocta-2,6-dien-1-yl)oxy)benzaldehyde* (**14**)

Compound **14** was obtained from 4-hydroxybenzaldehyde (1.0 g, 8.2 mmol) and geranyl bromide (2.3 g, 11 mmol) in DMF (20 mL). Compound **14** was isolated as a pale-yellow oil (1.9 g, 91% yield). ^1^H NMR (400 MHz, CDCl_3_): δ 9.84 (s, 1H, –CHO); 7.80 (d, 1H, *J* = 8.7 Hz, H-2 and H-6); 6.98 (d, 1H, *J* = 8.7 Hz, H-3 and H-5); 5.45 (m, 1H, H-2′); 5.06 (m, 1H, H-6′); 4.60 (d, *J* = 6.6 Hz, 2H, H-1′); 2.10–2.07 (m, 4H, H-5′ and H-4′); 1.73 (s, 3H, H-8′); 1.65 (s. 3H, CH_3_-C3′); 1.58 (s, 3H, CH_3_-C7′). ^13^C NMR (100 MHz, CDCl_3_): δ 190.7 (C=O); 163.9 (C-4); 142.0 (C-3′); 131.9 (C-2 and C-6); 131.9 (C-7′); 129.8 (C-1); 123.7 (C-6′); 118.7 (C-2′); 115.0 (C-3 and C-5); 65.2 (C-1′); 39.5 (C-4′); 26.2 (C-5′); 25.7 (C-8′); 17.7 (CH_3_-C7′); 16.7 (CH_3_-C3′). HRMS (ESI^+^): *m*/*z* calcd. for C_17_H_22_O_2_ [M + H]^+^: 259.1693; found: 259.1691. (see Figure S51, Supplementary Material).

*(E)-4-((3,7-dimethylocta-2,6-dien-1-yl)oxy)-3,5-dimethoxybenzaldehyde* (**15**)

Compound **15** was obtained from 4-hydroxy-3,5-dimethoxybenzaldehyde (1.0 g, 5.5 mmol) and geranyl bromide (1.4 g, 6.6 mmol) in DMF (20 mL). Compound **15** was isolated as a pale-yellow oil (1.5 g, 87% yield). ^1^H NMR (400 MHz, CDCl_3_): δ 9.54 (s, 1H, –CHO); 6.83 (s, 2H, H-4 and H-6); 5.27 (m, 1H, H-2′); 4.76 (m, 1H, H-6′); 4.36 (d, *J* = 7.3 Hz, 2H, H-1′); 3.61 (s, 6H, CH_3_O-C3 and CH_3_O-C5); 1.77–1.73 (m, 4H, H-5′ and H-4′); 1.39 (s, 3H, H-8′); 1.37 (s. 3H, CH3-C3′); 1.30 (s, 3H, CH3-C7′). ^13^C NMR (100 MHz, CDCl_3_): δ 190.6 (C=O); 153.9 (C-3 and C-5); 138.1 (C-1); 142.1 (C-4); 141.6 (C-3′); 131.6 (C-1); 131.1 (C-7′); 123.7 (C-6′); 119.8 (C-2′); 106.2 (C-2 and C-6); 69.0 (C-1′); 55.8 (2 x OCH_3_); 39.7 (C-4′); 26.1 (C-5′); 25.3 (C-8′); 17.3 (CH_3_-C7′); 16.0 (CH_3_-C3′). HRMS (ESI^+^): *m*/*z* calcd. for C_19_H_26_O_4_ [M + H]^+^: 319.1904; found: 319.1904. (see Figure S52, Supplementary Material).

*(E)-4-((3,7-dimethylocta-2,6-dien-1-yl)oxy)-3-methoxybenzaldehyde* (**16**)

Compound **16** was obtained from 4-hydroxy-3-methoxybenzaldehyde (1.0 g, 6.6 mmol) and geranyl bromide (1.7 g, 7.9 mmol) in DMF (20 mL). Compound **16** was isolated as a pale-yellow oil (1.8 g, 95% yield). ^1^H NMR (400 MHz, CDCl_3_): δ 9.83 (s, 1H, –CHO); 7.40 (m, 2H, H-2 and H-6); 6.95 (d, *J* = 8.1 Hz, 1H, H-5); 5.49 (m, 1H, H-2′); 5.05 (m, 1H, H-6′); 4.70 (d, *J* = 6.6 Hz, 2H, H-1′); 3.92 (s, 3H, CH_3_O-C3); 2.10–2.06 (m, 4H, H-5′ and H-6′); 1.74 (s, 3H, H-8′); 1.64 (s. 3H, CH_3_-C3′); 1.58 (s, 3H, CH_3_-C7′). ^13^C NMR (100 MHz, CDCl_3_): δ 190.9 (C=O); 153.9 (C-4); 150.0 (C-3); 141.7 (C-3′); 131.9 (C-7′); 129.1 (C-1); 126.8 (C-6); 123.7 (C-6′); 118.8 (C-2′); 111.7 (C-2); 109.1 (C-5); 66.1 (C-1′); 56.0 (OCH_3_); 39.5 (C-4′); 26.2 (C-5′); 25.8 (C-8′); 17.7 (CH_3_-C7′); 16.8 (CH_3_-C3′). HRMS (ESI^+^): *m*/*z* calcd. for C_18_H_24_O_3_ [M + H]^+^: 289.1798; found: 289.1789. (see Figure S53, Supplementary Material).

*(E)-4-((3,7-dimethylocta-2,6-dien-1-yl)oxy)-2-hydroxybenzaldehyde* (**17**) *and 2,4-bis(((E)-3,7-dimethylocta-2,6-dien-1-yl)oxy)benzaldehyde* (**20**)

Compounds **17** and **20** were synthesized from 2,4-dihydroxybenzaldehyde (1 g, 7.3 mmol) and geranyl bromide (1.9 g, 8.7 mmol) in DMF (20 mL). Two fractions were obtained by C.C. Fraction I: Compound **17** as a pale-yellow oil (560 mg, 28% yield). ^1^H NMR (400 MHz, CDCl_3_): δ 11.5 (s, 1H, OH); 9.7 (s, 1H, –CHO); 7.41 (d, 1H, *J* = 8.6 Hz, H-6); 6.54 (dd, *J* = 8.6 and 2.3 Hz, 1H, H-5); 6.42 (d, 1H, *J* = 2.3 Hz, H-3); 5.50 (m, 1H, H-2′); 5.08 (m, 1H, H-6′); 4.59 (d, *J* = 6.7 Hz, 2H, H-1′); 2.14–2.07 (m, 4H, H-5′ and H-4′); 1.74 (s, 3H, H-8′); 1.67 (s. 3H, CH3-C3′); 1.61 (s, 3H, CH3-C7′). ^13^C NMR (100 MHz, CDCl_3_): δ 194.3 (C=O); 166.2 (C-4); 164.5 (C-2); 142.4 (C-3′); 135.2 (C-6); 132.0 (C-7′); 123.6 (C-6′); 118.3 (C-2′); 115.1 (C-1); 109.0 (C-5); 101.3 (C-3); 65.4 (C-1′); 39.5 (C-4′); 26.2 (C-5′); 25.7 (C-8′); 17.7 (CH_3_-C7′); 16.7 (CH_3_-C3′). HRMS (ESI^+^): *m*/*z* calcd. for C_17_H_22_O_3_ [M + H]^+^: 275.1642; found: 275.1649. (see Figure S54, Supplementary Material). Fraction II: Compound **20** (950 mg, 32% yield) obtained as a pale-yellow oil). ^1^H NMR (400 MHz, CDCl_3_): δ 10.3 (s, 1H, –CHO); 7.79 (d, 1H, *J* = 8.6 Hz, H-6); 6.53 (dd, *J* = 8.6 and 2.2 Hz, 1H, H-4); 6.46 (d, 1H, *J* = 2.2 Hz, H-3); 5.47 (m, 1H, H-2′ and H-2″); 5.08 (m, 1H, H-6′ and H-6″); 4.60 (d, *J* = 6.9 Hz, 2H, H-1′); 4.59 (d, *J* = 6.9 Hz, 2H, H-1″); 2.13–2.07 (m, 8H, H-5′, H-4′, H-5″ and H-4″); 1.74 (s, 3H, H-8′); 1.73 (s, 3H, H-8″); 1.67 (s. 6H, CH_3_-C3′ and CH_3_-C3″); 1.60 (s, 6H, CH_3_-C7′ and CH_3_-C7″). ^13^C NMR (100 MHz, CDCl_3_): δ 188.5 (C=O); 165.4 (C-4); 163.1 (C-2); 142.1* (C-3′); 141.8* (C-3″); 131.9 (C-7′ and C-7″); 130.2 (C-6); 123.6 (C-6′ and C-6″); 119.2 (C-1); 118.8** (C-2′); 118.6** (C-2″); 106.5 (C-5); 99.6 (C-3); 65.5*** (C-1′); 65.2*** (C-1″); 39.5¤ (C-4′); 39.4¤ (C-4″); 26.3 (C-5′ and C-5″); 25.7 (C-8′ and C-8″); 17.7 (CH_3_-C7′ and CH_3_-C7″); 16.7 (CH_3_-C3′ and CH_3_-C3″). HRMS (ESI^+^): *m*/*z* calcd. for C_27_H_38_O_3_ [M + H]^+^: 411.2894; found: 411.2889. * ** *** ¤: interchangeable signals. (see Figure S57, Supplementary Material).

*(E)-2-((3,7-dimethylocta-2,6-dien-1-yl)oxy)benzaldehyde* (**18**)

Compound **18** was synthesized from 2-hydroxybenzaldehyde (1 g, 8.2 mmol) and geranyl bromide (2.14 g, 9.8 mmol) in DMF (20 mL). The reaction afforded compound **18** as a pale-yellow oil (1.97 g, 93% yield). ^1^H NMR (400 MHz, CDCl_3_): δ 10.5 (s, 1H, –CHO); 7.84 (dd, 1H, *J* = 7.7 and 1.8 Hz, H-6); 7.52 (m, 1H, H-4); 7.00 (m, 2H, H-3 and H-5); 5.50 (m, 1H, H-2′); 5.09 (m, 1H, H-6′); 4.67 (d, *J* = 6.5 Hz, 2H, H-1′); 2.13–2.10 (m, 4H, H-5′ and H-4′); 1.76 (s, 3H, H-8′); 1.68 (s, 3H, CH_3_-C3′); 1.61 (s, 3H, CH_3_-C7′). ^13^C NMR (100 MHz, CDCl_3_): δ 189.9 (C=O); 161.4 (C-2); 141.8 (C-3′); 135.8 (C-4); 131.9 (C-7′); 128.2 (C-6); 125.1 (C-1); 123.6 (C-6′); 120.5 (C-5); 118.9 (C-2′); 113.0 (C-3); 65.5 (C-1′); 39.5 (C-4’); 26.2 (C-5′); 25.7 (C-8′); 17.7 (CH_3_-C7′); 16.7 (CH_3_-C3′). HRMS (ESI^+^): *m*/*z* calcd. for C_17_H_22_O_2_ [M + H]^+^: 259.1693; found: 259.1691. (see Figure S55, Supplementary Material).

*(E)-2-((3,7-dimethylocta-2,6-dien-1-yl)oxy)-5-hydroxybenzaldehyde* (**19**)

Compound **19** was synthesized from 2,5-dihydroxybenzaldehyde (1 g, 7.3 mmol) and geranyl bromide (1.89 g, 8.7 mmol) in DMF (20 mL). The reaction afforded compound **19** as a pale-yellow oil (1.15 g, 58% yield). ^1^H NMR (400 MHz, CDCl_3_): δ 10.4 (s, 1H, –CHO); 7.37 (d, 1H, *J* = 7.3 Hz, H-6); 7.11 (dd, *J* = 8.9 and 3.2 Hz, 1H, H-4); 6.90 (d, 1H, *J* = 8.9 Hz, H-3); 5.47 (m, 1H, H-2′); 5.07 (m, 1H, H-6′); 4.60 (d, *J* = 6.6 Hz, 2H, H-1′); 2.10–2.05 (m, 4H, H-4′ and H-5′); 1.72 (s, 3H, H-8′); 1.67 (s. 3H, CH_3_-C3′); 1.60 (s, 3H, CH_3_-C7′). ^13^C NMR (100 MHz, CDCl_3_): δ 190.7 (C=O); 156.0 (C-2); 150.0 (C-5); 141.8 (C-3′); 131.9 (C-7′); 125.4 (C-1); 123.8 (C-4); 123.6 (C-6′); 118.9 (C-2′); 115.2 (C-3); 113.3 (C-6); 66.3 (C-1′); 39.4 (C-4′); 26.2 (C-5′); 25.6 (C-8′); 17.7 (CH_3_-C7′); 16.7 (CH_3_-C3′). HRMS (ESI^+^): *m*/*z* calcd. for C_17_H_22_O_3_ [M + H]^+^: 275.1642; found: 275.1645. (see Figure S56, Supplementary Material).

#### 3.2.3. Antifungal Activity Evaluation

The fungitoxic activity of the geranylated benzaldehydes was evaluated by determining radial growth inhibition on potato dextrose agar (PDA) solid medium. Stock solutions of the compounds were prepared in dichloromethane to achieve target concentrations of 20, 40, 80, and 160 µg/mL for Botrytis cinerea, and 3, 6, 12, 25, and 50 µg/mL for Phytophthora cinnamomi. An aliquot of each solution (800 µL) was incorporated into 25 mL of molten PDA. The final concentration of dichloromethane remained constant across all control and treatment assays.

To evaporate the solvent, the mixture was stirred continuously in a water bath at 50 °C for 2 min. Subsequently, 7 mL of the medium was poured into 60 mm diameter Petri dishes. The plates were left open in a biosafety cabinet under ultraviolet light for 40 min to dry and sterilize. The Petri dishes were then inoculated with 6 mm agar plugs cut from the active margin of mycelial cultures of either B. cinerea or P. cinnamomi. Cultures were incubated in the dark at 22 °C for 3 days. Mycelial growth diameter was measured using a digital caliper, and photographs were recorded at 48 h. All assays were performed in triplicate.

Mycelial growth inhibition rates were expressed as percentages (%) and calculated using the following equation:

$$Growth\;inhibition\;rate\;\left(\%\right):\;\frac{\left(d_{C}-d_{0}\right)-(d_{S}-d_{0})}{d_{C}-d_{0}}\;\times \;100$$

where *d*_0_ is the diameter of the inoculated agar plug (6 mm), *d*_C_ is the mean colony diameter of the negative control, and *d*_S_ is the colony diameter of the compound-treated sample. The half-maximal effective concentration (EC_50_) values for each compound against *B. cinerea* and *P. cinnamomi* were calculated by non-linear regression analysis using a four-parameter sigmoidal dose–response model. Values were expressed in µg/mL.

#### 3.2.4. Statistical Analysis

All data were expressed as the mean ± standard deviation (SD). Due to non-parametric data, a Kruskal–Wallis ANOVA was used with STATISTIC 7.0 program. Statistical significance was defined as *p* < 0.05.

### 3.3. Molecular Docking

The crystal structure of succinate dehydrogenase (SDH, PDB ID: 2FBW) was prepared at pH 7.5 using the PDB2PQR web server PROPKA (3.1) and UCSF Chimera (v1.16), removing crystallographic waters while retaining the prosthetic heme (HEM) group. Ligand 3D structures were energy-minimized using the MMFF94 force field. Docking calculations were performed in AutoDock Vina (v1.1.2) with a grid box centered on the ubiquinone Q-site near the HEM group utilizing a grid box of dimensions 20 × 20 × 20 Å; exhaustiveness = 16. The lowest binding free energy poses (kcal/mol) were selected, and non-covalent interactions were analyzed using UCSF Chimera [60,61] and Discovery Studio Visualizer v21.1 [62].

## 4. Conclusions

In this study, a series of geranylated benzaldehydes was successfully designed and synthesized using C–C and C–O coupling strategies. The chemical structures of all derivatives were unequivocally elucidated by comprehensive ^1^H and ^13^C NMR analyses, supported by 2D NMR techniques (HSQC and HMBC), enabling clear differentiation between C–C and C–O geranyl linkages.

The antifungal evaluation against *Botrytis cinerea* and *Phytophthora cinnamomi* revealed a species-dependent response. Among the tested compounds, derivative **19** emerged as the most promising displaying moderate inhibition against *B. cinerea* and enhanced potency against *P. cinnamomi*, as reflected by its inhibition percentages and EC_50_ values. These findings indicate that subtle structural features, including the mode of geranyl attachment and the pattern of aromatic substitution, play a key role in modulating antifungal activity.

Molecular docking studies targeting succinate dehydrogenase (SDH) provided further insight into a potential mode of action for the most active compounds. Compound **19** exhibited the most favorable predicted binding affinity, which may be associated with the spatial arrangement of its functional groups within the binding pocket. The predicted hydrogen-bonding, hydrophobic, and π–cation interactions may contribute to ligand stabilization and are consistent with the superior antifungal activity observed for this compound.

Although the obtained results demonstrate antifungal activity based on mycelial growth inhibition assays, they should be interpreted within the context of the experimental approach employed. The biological evaluation was conducted using in vitro measurements of radial mycelial growth, providing an initial assessment of compound activity. While molecular docking offers valuable insights into potential target interactions, further studies are required to gain a deeper understanding of the mode of action, including enzymatic assays targeting SDH to validate the proposed mechanism. In addition, factors such as compound stability, bioavailability, and plant–pathogen interactions, as well as in planta evaluations, should be considered to achieve a more comprehensive assessment of their biological potential. Overall, these findings provide a solid foundation for future studies aimed at validating and expanding upon these results.

## Figures and Tables

**Figure 1:**
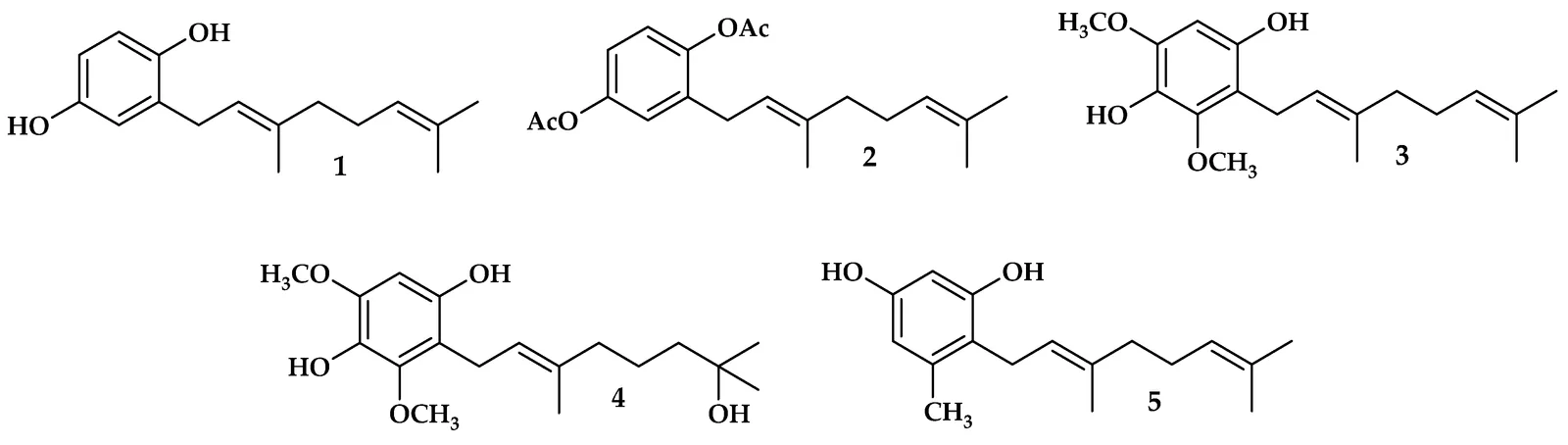
Representative naturally occurring geranylated aromatic compounds (**1**–**5**) with reported biological activities.

**Figure 2:**
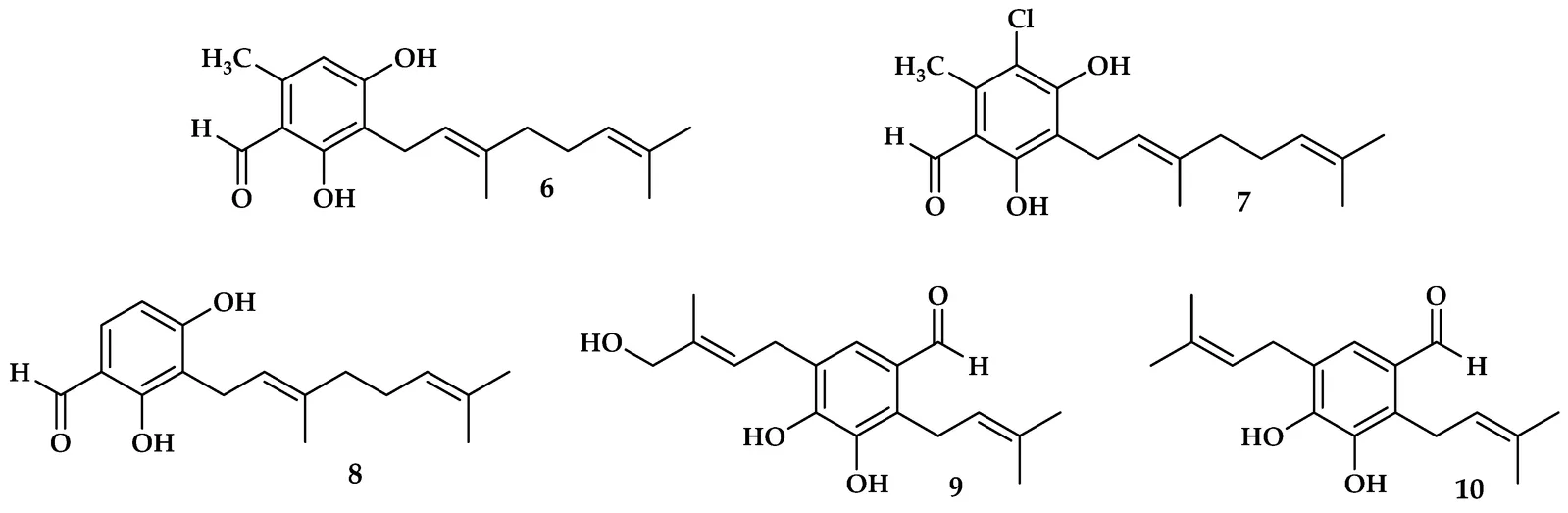
Examples of synthetic bioactive prenylated aromatic compounds (**6**–**10**) reported in the literature that inspired the design of the present study.

**Figure 3:**
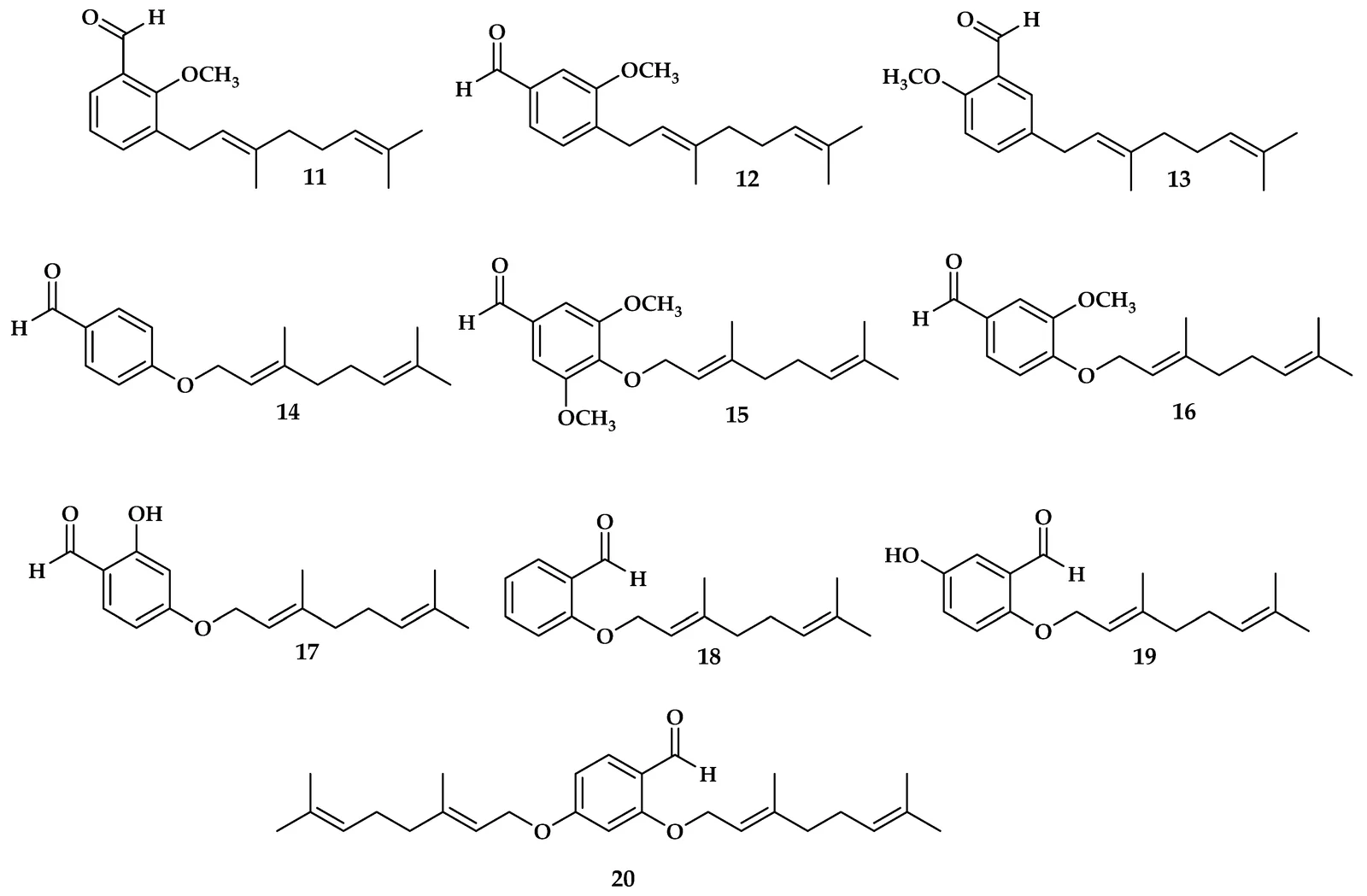
Geranylated benzaldehydes with *C*–*C* (**11**–**13**) or *C*–*O* bonds (**14**–**20**).

**Scheme 1:**
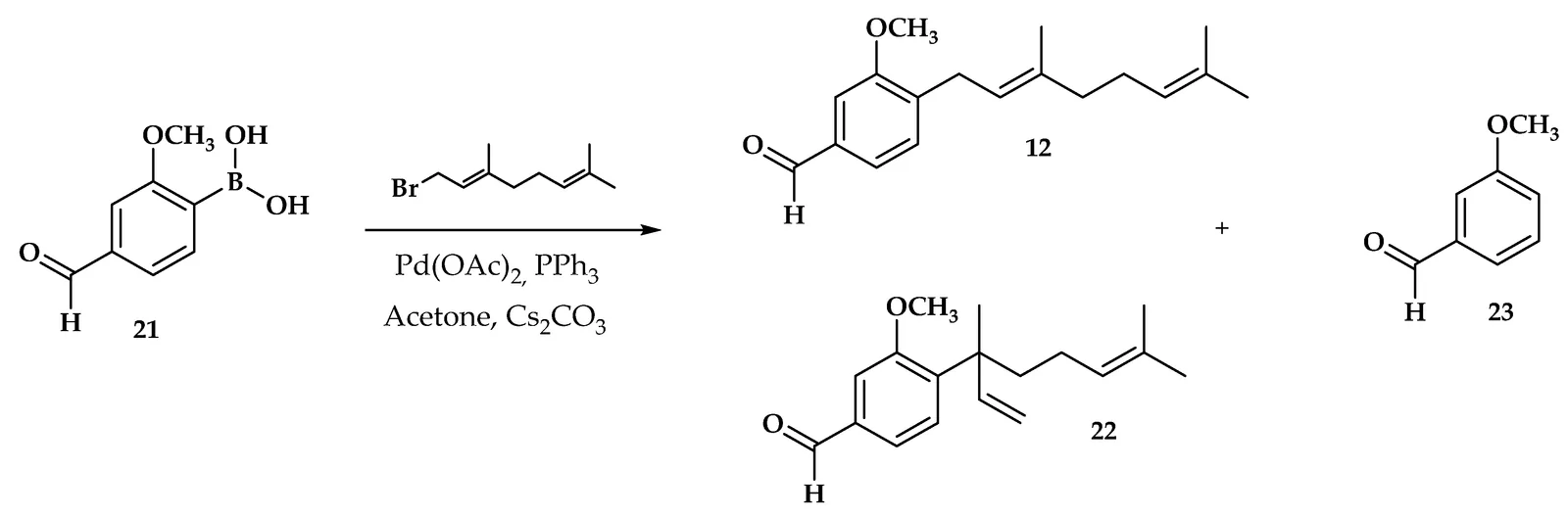
Synthesis of *C*-geranylated benzaldehyde **12**, its isomer **22** and a byproduct **23**.

**Scheme 2:**
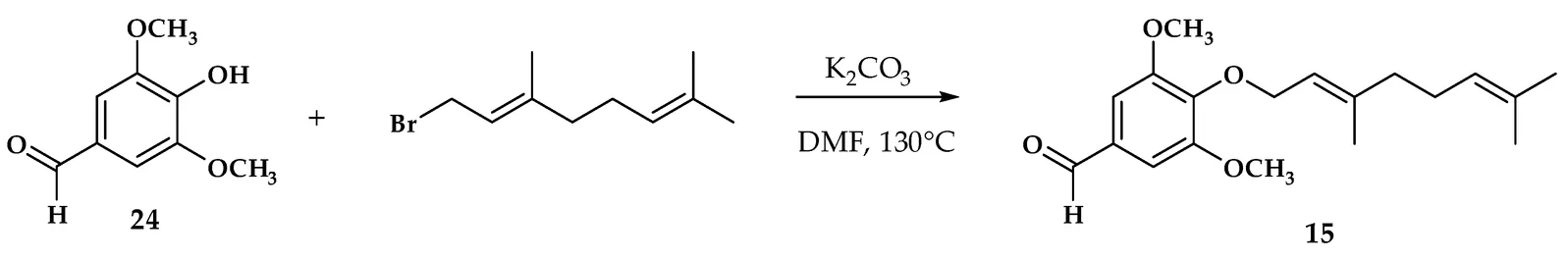
Synthesis of *O*-geranylated benzaldehyde **15**.

**Figure 4:**
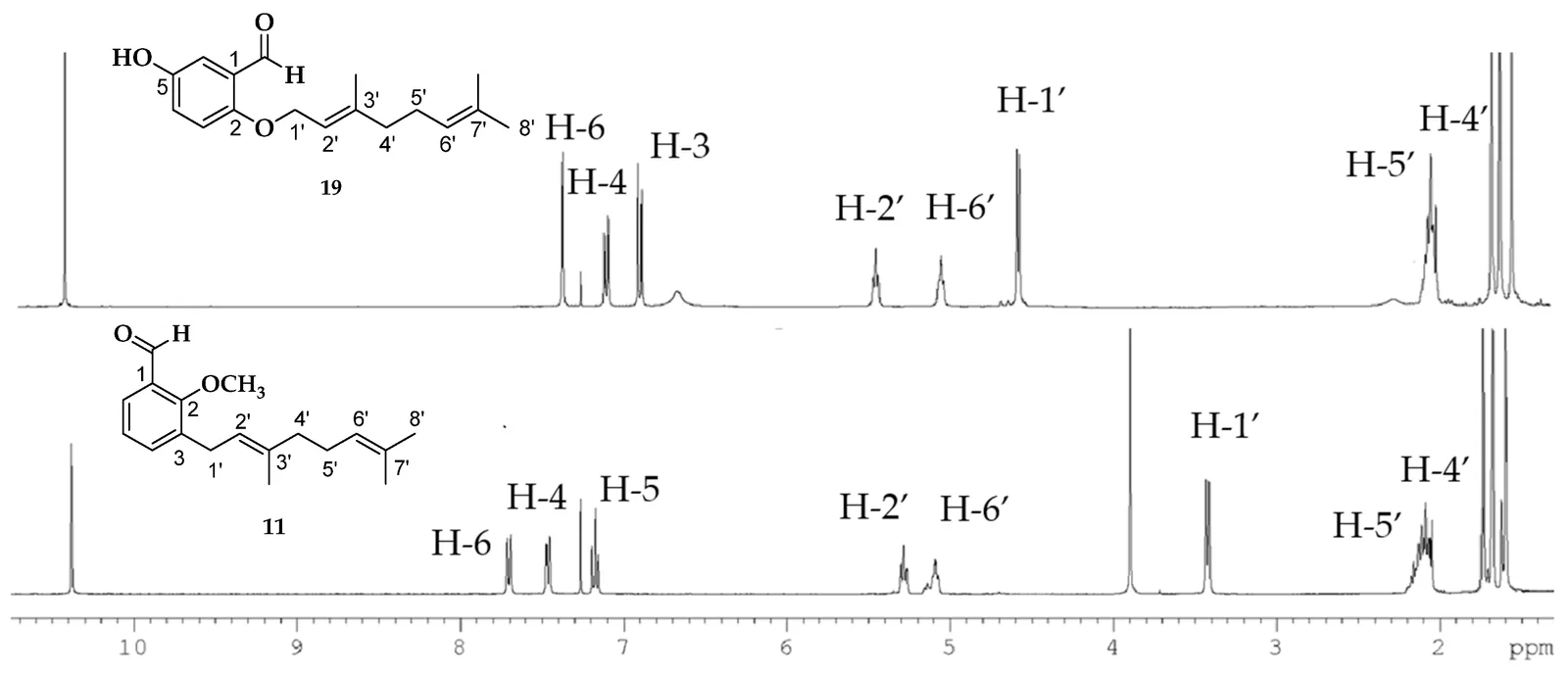
Comparison of the ^1^H NMR spectra of C–O-coupled geranylated benzaldehyde **19** and C–C-coupled geranylated benzaldehyde **11**.

**Figure 5:**
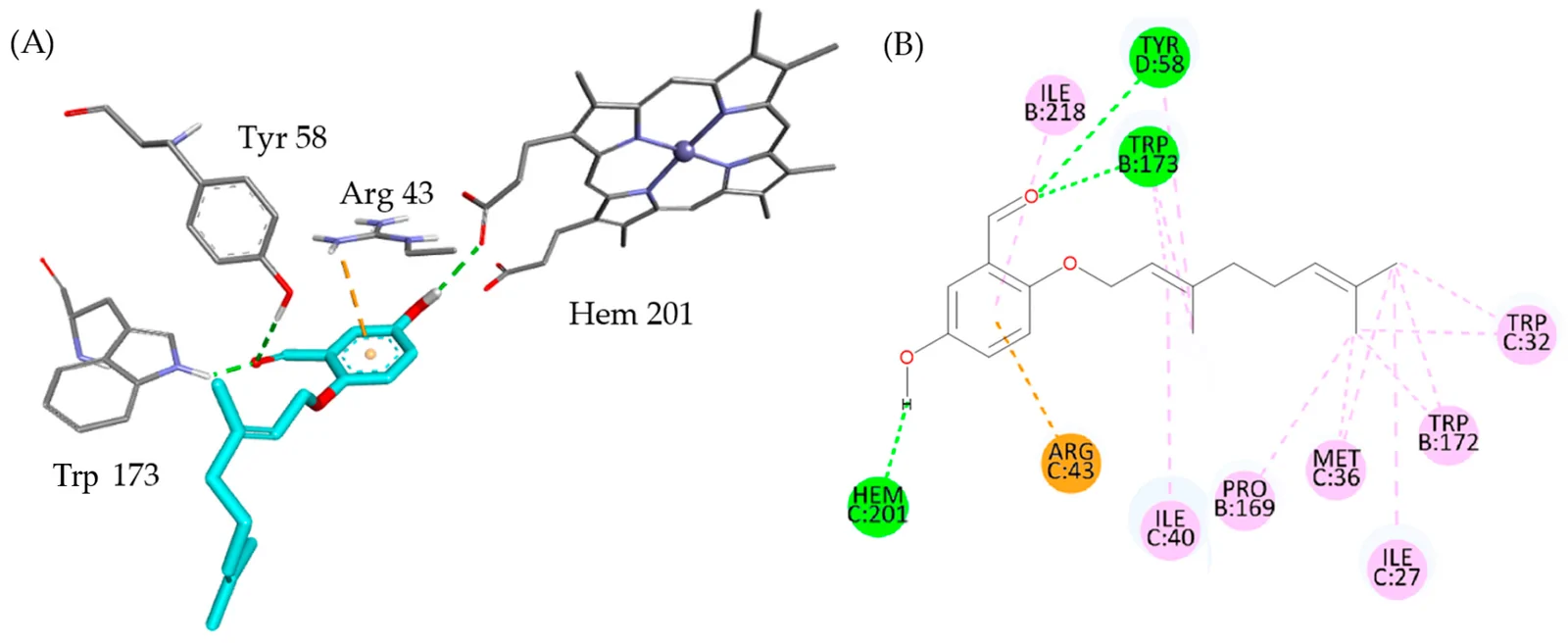
Predicted binding mode of **19** into the allosteric site of enzyme SDH (PDB 2FBW). (**A**) pose of **19** and its interaction within the SDH binding site; (**B**) a 2D view of binding interactions of **19** within the binding site. Hydrogen bonds are represented as green dotted lines. π-cation is represented in orange segmented lines. Hydrophobic interactions are represented in pink segmented lines.

**Table 1:** Percentages of growth inhibition of *B. cinerea* by geranylated benzaldehydes **11**–**20**.

*B. cinerea* Percentage of Growth Inhibition (%)					
Compound	10 µg/mL	20 µg/mL	40 µg/mL	80 µg/mL	160 µg/mL
**11**	0	9.7 ± 3.0	7.8 ± 7.0	13.1 ± 7.4	18.1 ± 4.6
**12**	0	14.6 ± 12.0	6.3 ± 5.0	32.5 ± 5.3	23.0 ± 9.7
**13**	11.5 ± 6.7	10.8 ± 6.3	11.3 ± 3.0	14.2 ± 4.3	26.2 ± 4.8
**14**	5.0 ± 2.0	5.5 ± 0.8	5.4 ± 0.6	8.8 ± 2.8	10.9 ± 2.0
**15**	16.4 ± 13.3	21.1 ± 10.8	15.4 ± 2.2	32.9 ± 3.1	74.0 ± 7.5
**16**	0	8.2 ± 6.2	5.3 ± 1.3	12.7 ± 5.0	16.5 ± 2.7
**17**	0	0	1.9 ± 0.9	11.2 ± 1.1	16.8 ± 0.2
**18**	0	2.5 ± 4.6	4.8 ± 1.1	18.9 ± 4.1	25.0 ± 1.4
**19**	26.2 ± 10.4	35.9 ± 14.6	39.7 ± 13.1	44.0 ± 10.1	62.2 ± 11.1
**20**	0	0	0	0	0

All data are the means of three independent replicates.

**Table 2:** Effect of geranylated benzaldehyde structure on *P. cinnamomi* growth inhibition.

Compound	*Phytophthora cinnamomi* Inhibition of Growth (%)				
	3 µg/mL	6 µg/mL	12 µg/mL	25 µg/mL	50 µg/mL
**11**	3.3 ± 2.0	3.8 ± 1.4	3.0 ± 0.6	4.2 ± 2.2	3.7 ±1.2
**12**	1.6 ± 0.6	2.6 ±1.2	2.6 ± 1.9	3.6 ± 1.5	5.2 ± 1.0
**13**	0	0	0.5 ± 0.3	2.9 ± 1.2	3.6 ± 1.3
**14**	2.4 ± 2.0	7.4 ± 2.1	8.9 ± 1.5	20.8 ± 2.5	34.7 ± 0.8
**15**	0	0	2.1 ± 1.6	6.2 ± 1.9	10.0 ± 4.0
**16**	1.7 ± 1.3	5.4 ± 0.8	8.3 ± 0.8	12.2 ± 1.2	23.8 ± 3.0
**17**	0	0	6.3 ± 3.1	19.7 ± 4.3	44.7 ± 2.0
**18**	0.9 ± 0.9	2.1 ± 2.0	3.0 ± 1.8	13.3 ± 1.9	25.4 ± 1.6
**19**	34.4 ± 10.0	56.8 ± 7.9	86.6 ± 5.9	89.3 ± 0.5	88.3 ± 3.8
**20**	1.6 ± 1.5	4.8 ± 1.9	4.2 ± 4.0	7.0 ± 3.9	15.1 ± 3.9

All data are the means of three independent replicates.

**Table 3:** Comparative Antifungal Activity of Commercial Fungicides and Compound **19** Against *Botrytis cinerea* and *Phytophthora cinnamomi*: A Literature Review.

Commercial Fungicide	EC_50_ (*B. cinerea*)	EC_50_ (*P. cinnamomi*)
boscalid	0.70 µg/mL [56]	
pyrimethanil	50.0 µg/mL [56]	
2-allylphenol	22.9 µg/mL [57]	155.0 µg/mL [58]
potassium phosphite		81.5 µg/mL [59]
metalaxyl		7.5 µg/mL [58]
Compound **19**	83.0 µg/mL	7.34 µg/mL

## Data Availability

The original contributions presented in this study are included in the article/Supplementary Material. Further inquiries can be directed to the corresponding authors.

## Supplementary Materials

The following supporting information can be downloaded at https://www.mdpi.com/article/10.3390/molecules31193481/s1. Figures S1–S47: NMR spectroscopic data (^1^H NMR, ^13^C NMR, ^13^C DEPT-135, HSQC, and HMBC spectra) of compounds **11**–**20**. Figures S48–S57: HRMS spectra of compounds **11**–**20**. Figures S60–S69: Protein–ligand interaction diagrams of compounds **11**–**20** within the SDH active site. Table S1: Molecular interactions of compounds **11**–**20** within the SDH active site. Table S2: Pose analysis of docked synthetic analogs (**11**–**20**), including binding energy (ΔEb, kcal/mol).
