# Supplementary material for: Design and Synthesis of Geranylated Benzaldehydes: Evaluation of Antifungal Activities Against Botrytis cinerea and Phytophthora cinnamomi
Source: Molecules. 2026 Sep 29;31(19):3481. doi: 10.3390/molecules31193481 (PMC13666287; doi:10.3390/molecules31193481)
Supplement: Supplementary file 1 [file molecules-31-03481-s001.zip › molecules-4543430-supplementary.pdf]

molecules

Article

Design and Synthesis of Geranylated Benzaldehydes: Evaluation of Antifungal Activities against *Botrytis cinerea* and *Phytophthora cinnamomi*

Supplementary Materials: The following supporting information can be downloaded at: https://www.mdpi.com/article/doi/s1. Figures S1-S47: NMR spectroscopic data (^{1}H NMR, ^{13}C NMR, ^{13}C DEPT-135, HSQC, and HMBC spectra) of compounds 11-20. Figures S48-S57: HRMS spectra of compounds 11-20. Figures S58-S67: Protein-ligand interaction diagrams of compounds 11-20 within the SDH active site. Table S1: Molecular interactions of compounds 11-20 within the SDH active site. Table S2: Pose analysis of docked synthetic analogs (11-20), including binding energy (ΔEb, kcal/mol).

Index

	Pag.
**Figure S1**. ^{1}H NMR spectrum compound **11**	4
**Figure S2**. ^{13}C NMR spectrum compound **11**	4
**Figure S3**. ^{13}C DEPT-135 NMR spectrum of **11**	4
**Figure S4**. 2D HSQC NMR spectrum of **11**	5
**Figure S5**. 2D HMBC spectrum compound **11**	5
**Figure S6**. ^{1}H NMR spectrum compound **12**	6
**Figure S7**. ^{13}C NMR spectrum compound **12**	6
**Figure S8**. ^{13}C DEPT-135 NMR spectrum of **12**	6
**Figure S9**. 2D HSQC NMR spectrum of **12**	7
**Figure S10**. 2D HMBC spectrum compound **12**	7
**Figure S11**. ^{1}H NMR spectrum compound **13**	8
**Figure S12**. ^{13}C NMR spectrum compound **13**	8
**Figure S13**. ^{1}H NMR spectrum compound **14**	9
**Figure S14**. ^{13}C NMR spectrum compound **14**	9
**Figure S15**. ^{13}C DEPT-135 NMR spectrum of **14**	9
**Figure S16**. 2D HSQC NMR spectrum of **14**	10
**Figure S17**. 2D HMBC spectrum compound **14**	10
**Figure S18**. ^{1}H NMR spectrum compound **15**	11
**Figure S19**. ^{13}C NMR spectrum compound **15**	11
**Figure S20**. ^{13}C DEPT-135 NMR spectrum of **15**	11
**Figure S21**. 2D HSQC NMR spectrum of **15**	12

**Figure S22**. 2D HMBC spectrum compound **15**	12
**Figure S23**. ^{1}H NMR spectrum compound **16**	13
**Figure S24**. ^{13}C NMR spectrum compound **16**	13
**Figure S25**. ^{13}C DEPT-135 NMR spectrum of **16**	13
**Figure S26**. 2D HSQC NMR spectrum of **16**	14
**Figure S27**. 2D HMBC spectrum compound **16**	14
**Figure S28**. ^{1}H NMR spectrum compound **17**	15
**Figure S29**. ^{13}C NMR spectrum compound **17**	15
**Figure S30**. ^{13}C DEPT-135 NMR spectrum of **17**	15
**Figure S31**. 2D HSQC NMR spectrum of **17**	16
**Figure S32**. 2D HMBC spectrum compound **17**	16
**Figure S33**. ^{1}H NMR spectrum compound **18**	17
**Figure S34**. ^{13}C NMR spectrum compound **18**	17
**Figure S35**. ^{13}C DEPT-135 NMR spectrum of **18**	17
**Figure S36**. 2D HSQC NMR spectrum of **18**	18
**Figure S37**. 2D HMBC spectrum compound **18**	18
**Figure S38**. ^{1}H NMR spectrum compound **19**	19
**Figure S39**. ^{13}C NMR spectrum compound **19**	19
**Figure S40**. ^{13}C DEPT-135 NMR spectrum of **19**	19
**Figure S41**. 2D HSQC NMR spectrum of **19**	20
**Figure S42**. 2D HMBC spectrum compound **19**	20
**Figure S43**. ^{1}H NMR spectrum compound **20**	21
**Figure S44**. ^{13}C NMR spectrum compound **20**	21
**Figure S45**. ^{13}C DEPT-135 NMR spectrum of **20**	21
**Figure S46**. 2D HSQC NMR spectrum of **20**	22
**Figure S47**. 2D HMBC spectrum compound **20**	22
**Figure S48.** HRSM spectrum of compound **11**	23
**Figure S49.** HRSM spectrum of compound **12**	23
**Figure S50.** HRSM spectrum of compound **13**	24
**Figure S51.** HRSM spectrum of compound **14**	24
**Figure S52.** HRSM spectrum of compound **15**	25
**Figure S53.** HRSM spectrum of compound **16**	25
**Figure S54.** HRSM spectrum of compound **17**	26
**Figure S55.** HRSM spectrum of compound **18**	26
**Figure S56.** HRSM spectrum of compound **19**	27
**Figure S57.** HRSM spectrum of compound **20**	27
**Figure S58.** In vitro effect of geranylated benzaldehyde (**19**) on mycelial growth of *B. cinerea* at different concentrations	28
**Figure S59.** In vitro effect of geranylated benzaldehyde (**19**) on mycelial growth of *P. cinnamomi* at different concentrations	28
**Figure S60.** Protein-ligand interactions with Compound **11**	28
**Figure S61.** Protein-ligand interactions with Compound **12**	29
**Figure S62.** Protein-ligand interactions with Compound **13**	29
**Figure S63.** Protein-ligand interactions with Compound **14**	29
**Figure S64.** Protein-ligand interactions with Compound **15**	30
**Figure S65.** Protein-ligand interactions with Compound **16**	30

**Figure S66.** Protein-ligand interactions with Compound **17**	31
**Figure S67.** Protein-ligand interactions with Compound **18**	31
**Figure S68.** Protein-ligand interactions with Compound **19**	32
**Figure S69.** Protein-ligand interactions with Compound **20**	32
**Table S1.** Molecular interactions of compounds **11–20** within the SDH active site.	33
**Table S2**. Pose analysis of docked synthetic analogs (**11-20**). ΔEb: Binding Energy in kcal/mol.	33

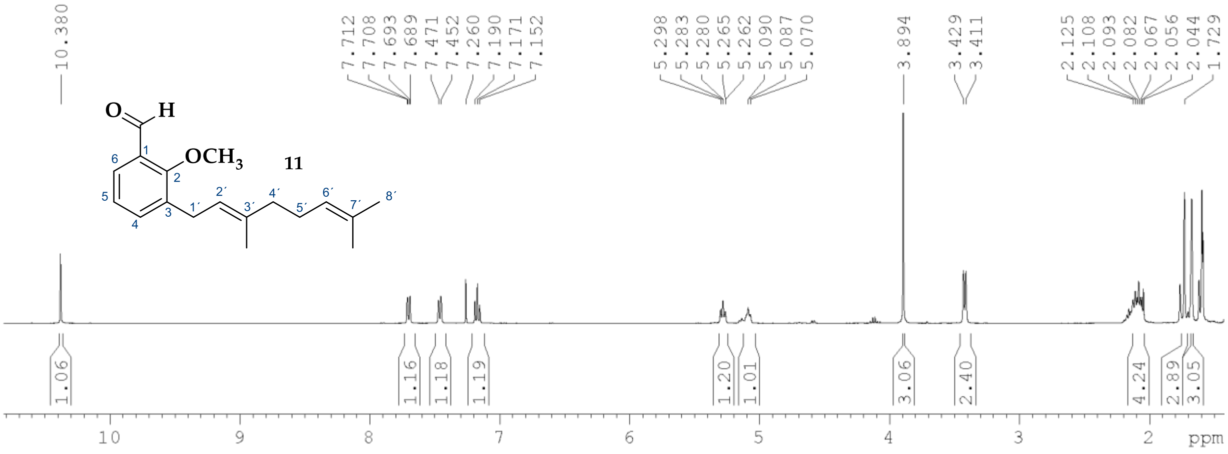

Figure S1. ^{1}H NMR spectrum compound **11**

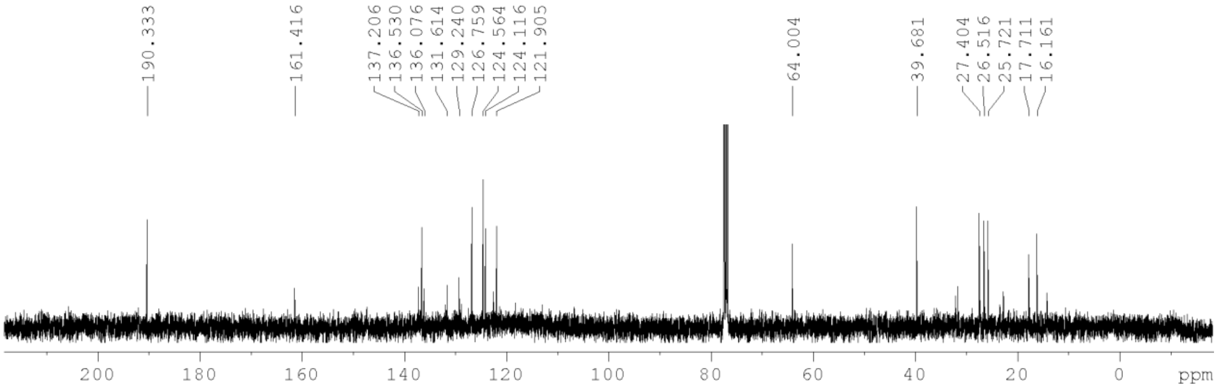

Figure S2. ^{13}C NMR spectrum compound **11**

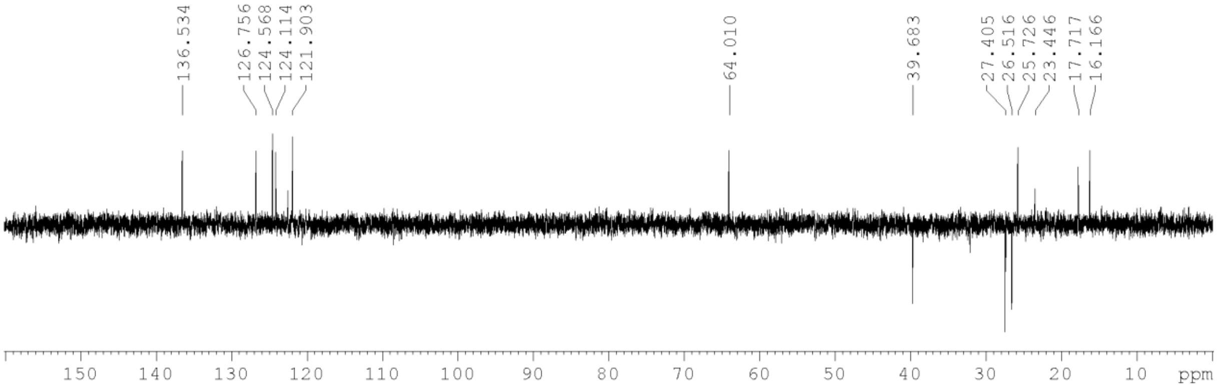

Figure S3. ^{13}C DEPT-135 NMR spectrum of **11**

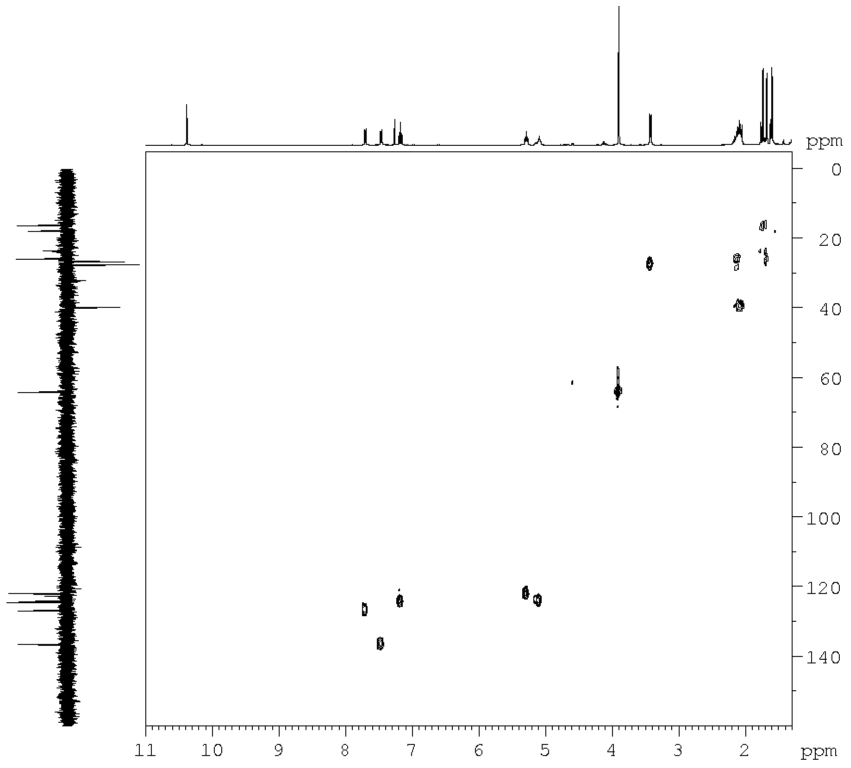

Figure S4. 2D HSQC NMR spectrum of **11**

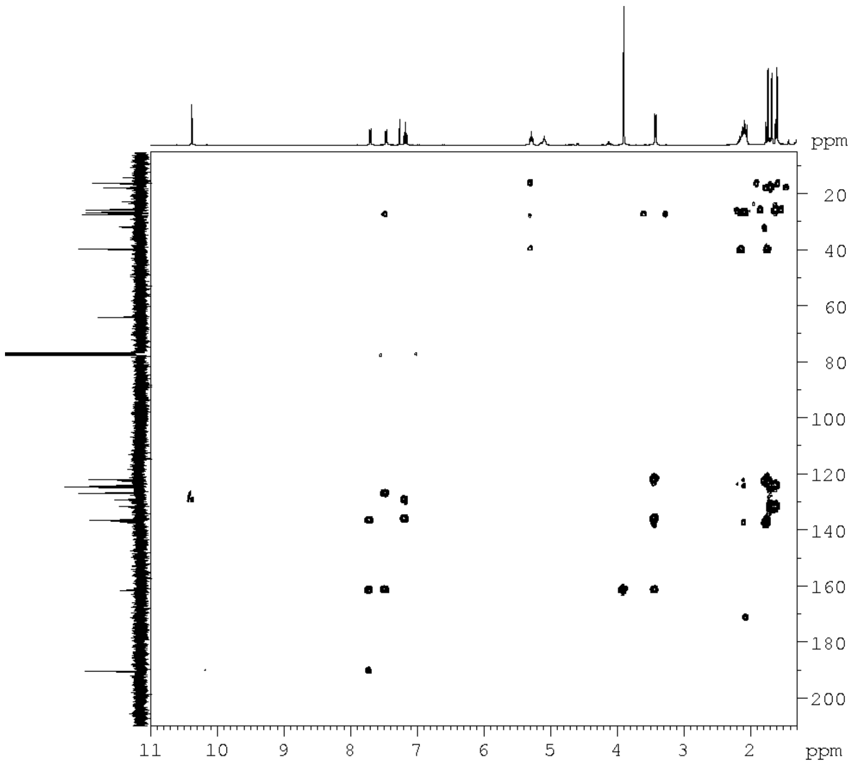

Figure S5. 2D HMBC spectrum compound **11**

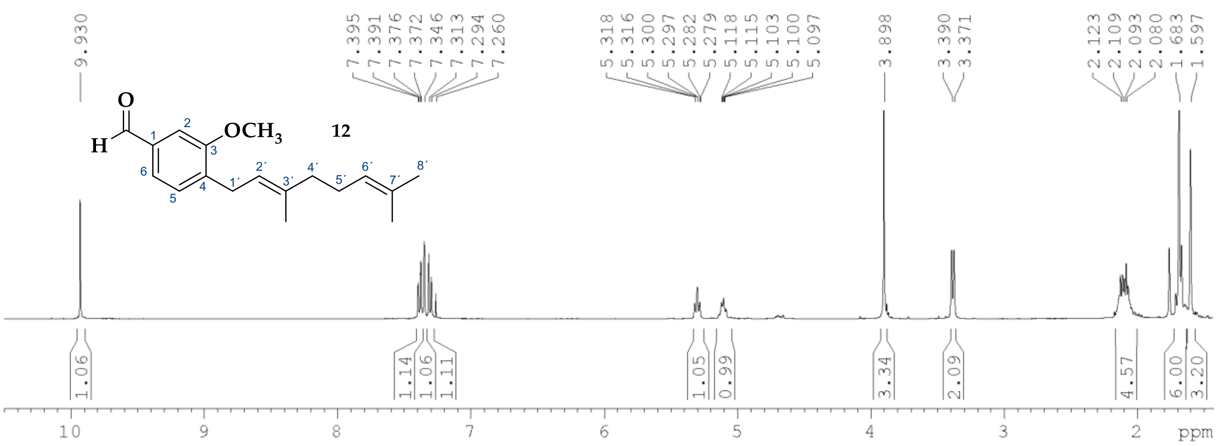

Figure S6. ^{1}H NMR spectrum compound **12**

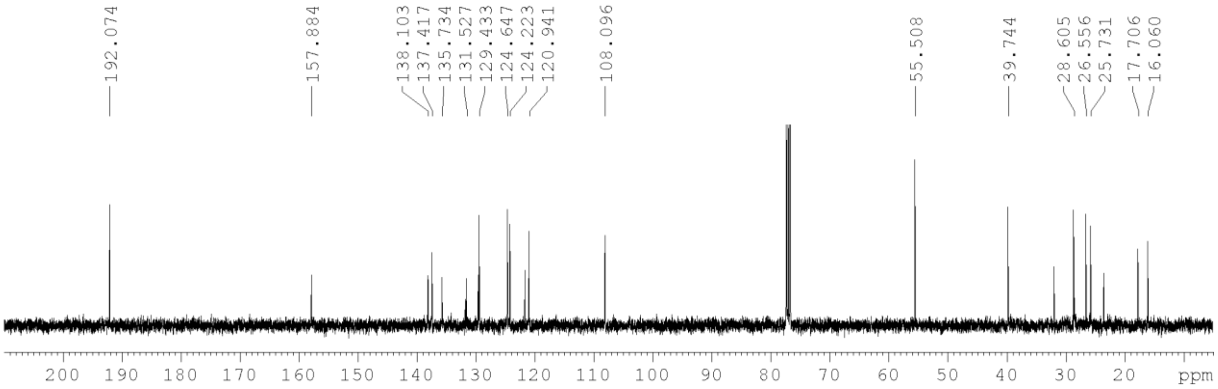

Figure S7. ^{13}C NMR spectrum compound **12**

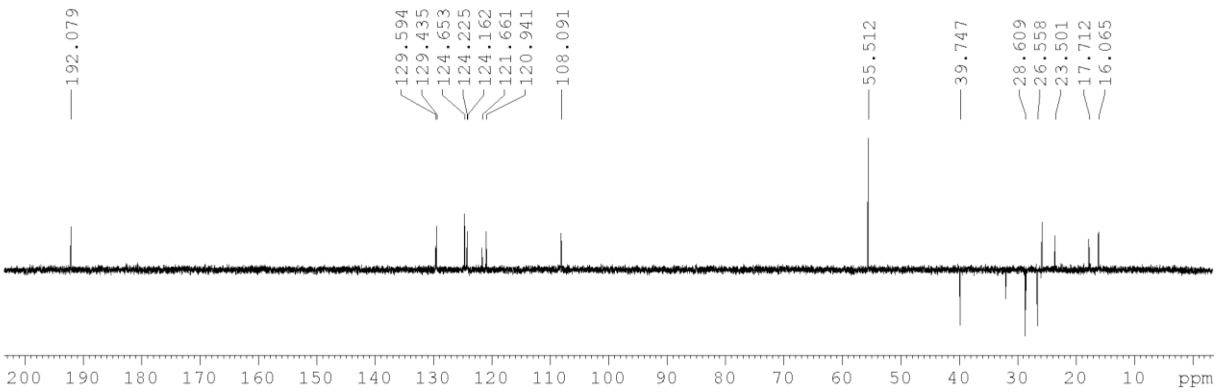

Figure S8. ^{13}C DEPT-135 NMR spectrum of **12**

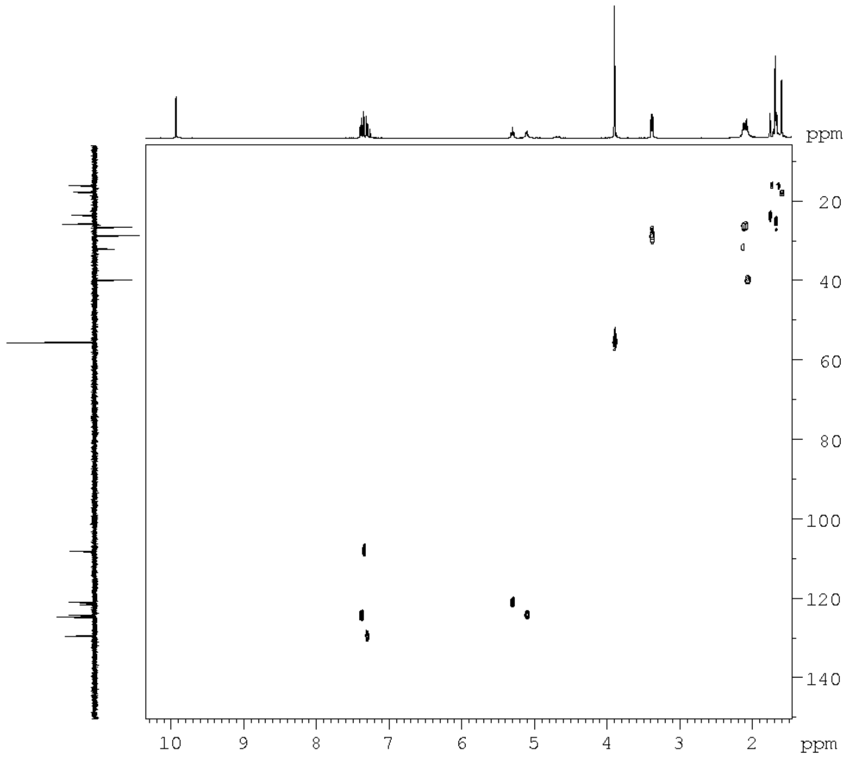

Figure S9. 2D HSQC NMR spectrum of **12**

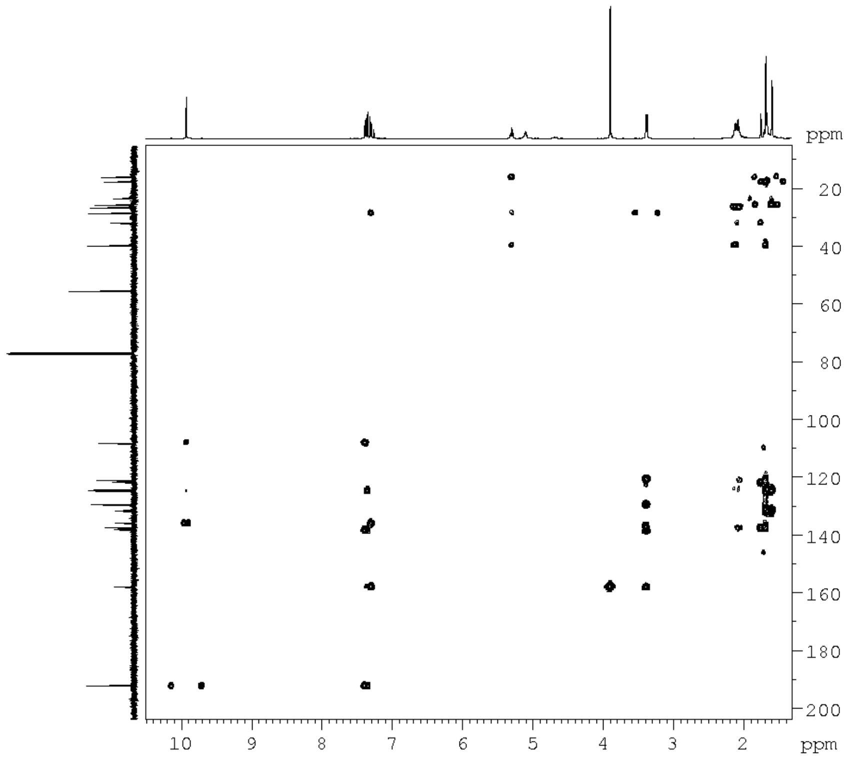

Figure S10. 2D HMBC spectrum compound **12**

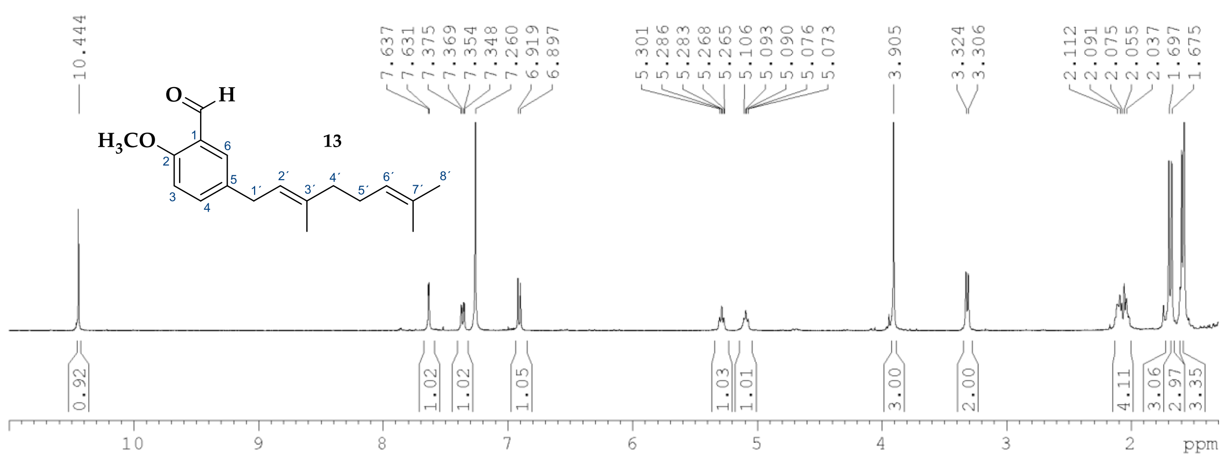

Figure S11. ^{1}H NMR spectrum compound **13**

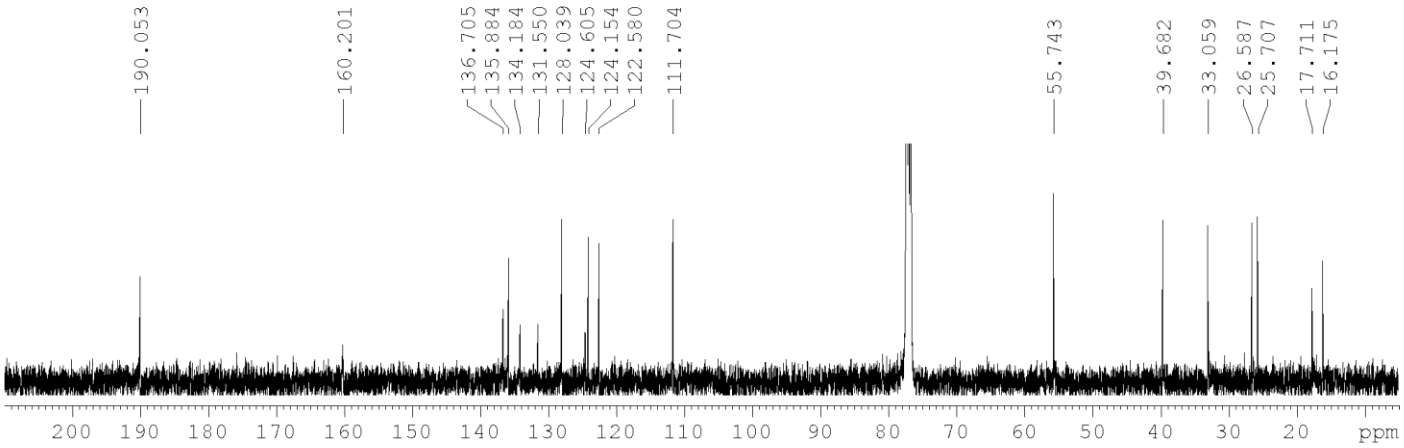

Figure S12. ^{13}C NMR spectrum compound **13**

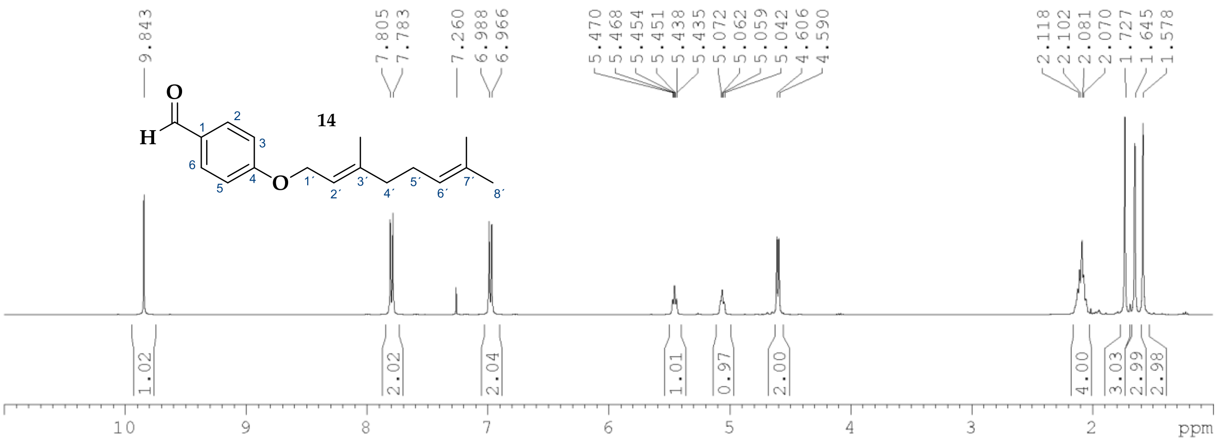

Figure S13. ^{1}H NMR spectrum compound **14**

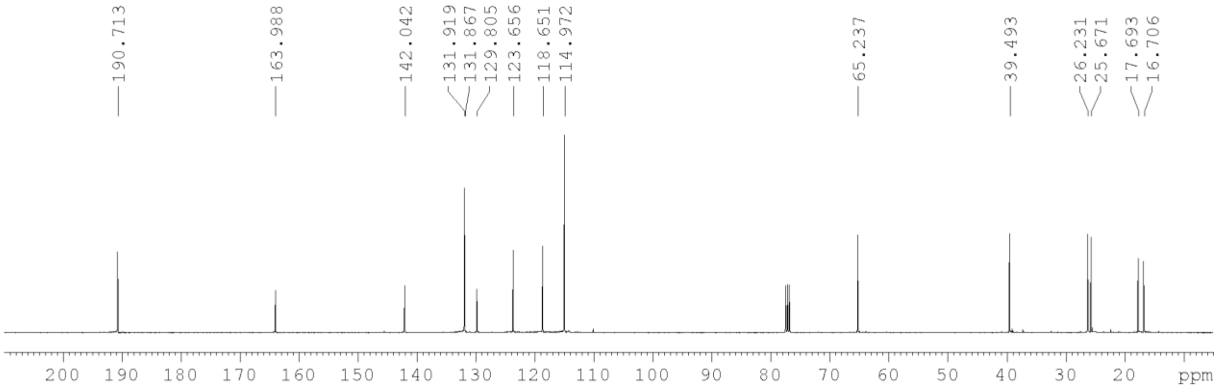

Figure S14. ^{13}C NMR spectrum compound **14**

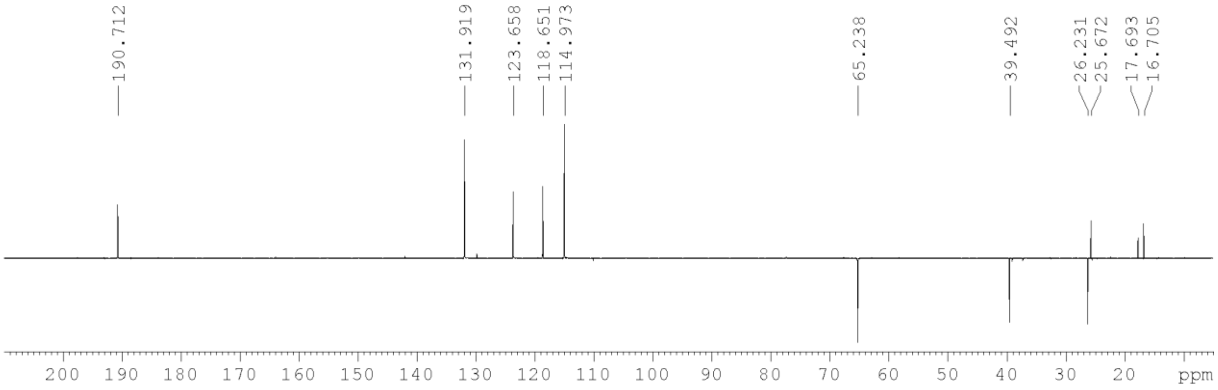

Figure S15. ^{13}C DEPT-135 NMR spectrum of **14**

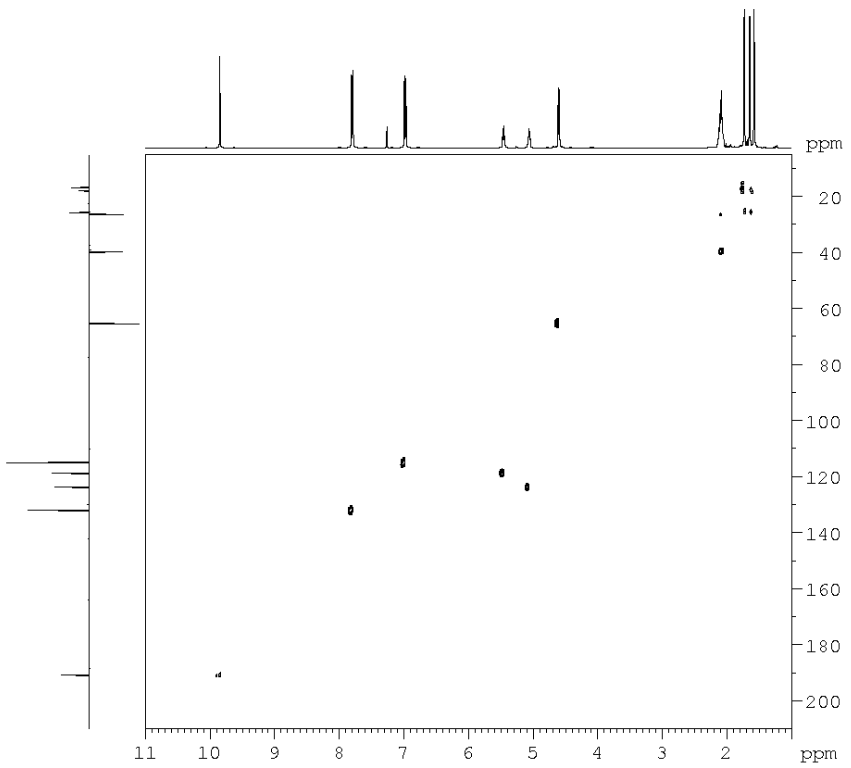

Figure S16. 2D HSQC NMR spectrum of **14**

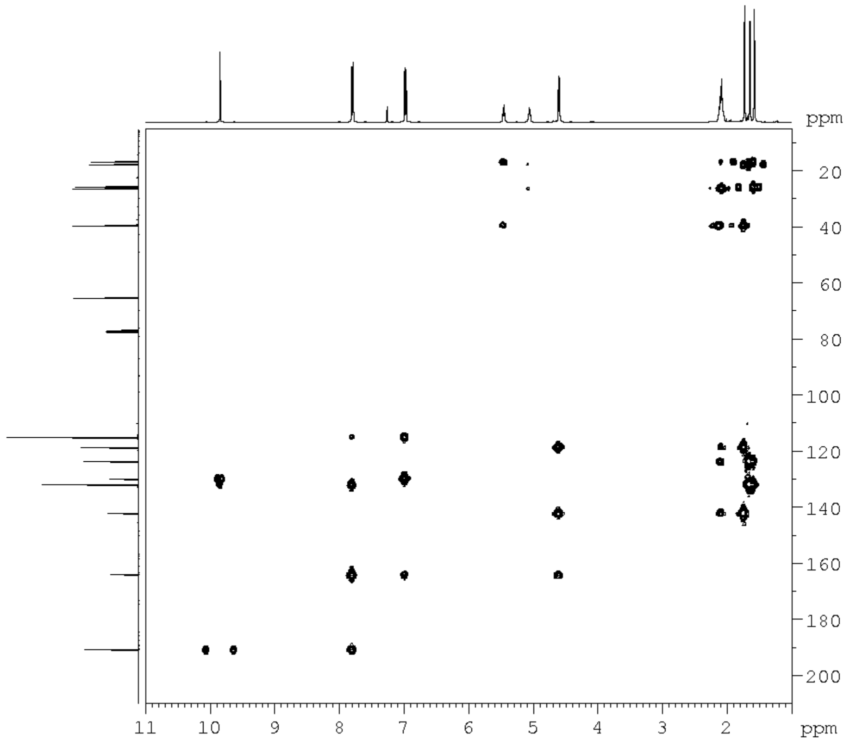

Figure S17. 2D HMBC spectrum compound **14**

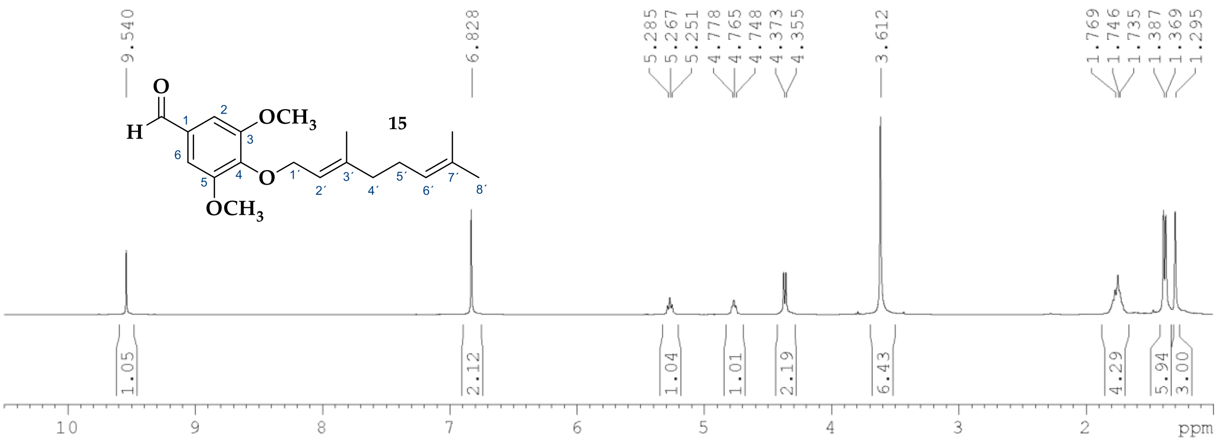

Figure S18. ^{1}H NMR spectrum compound **15**

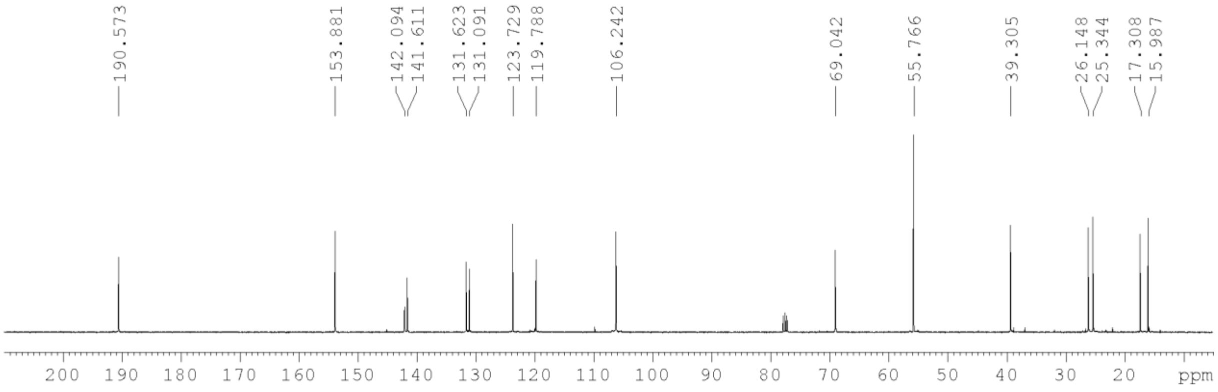

Figure S19. ^{13}C NMR spectrum compound **15**

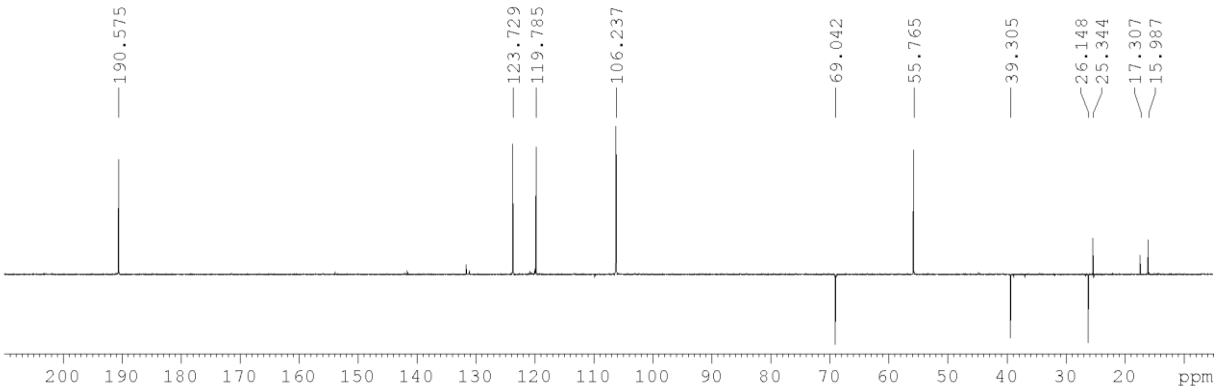

Figure S20. ^{13}C DEPT-135 NMR spectrum of **15**

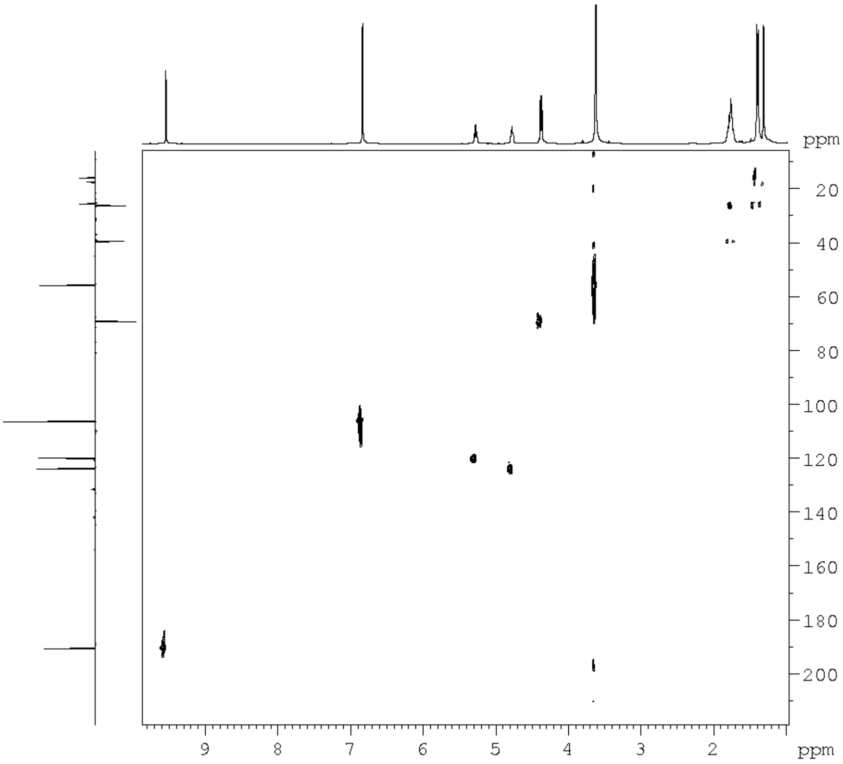

Figure S21. 2D HSQC NMR spectrum of **15**

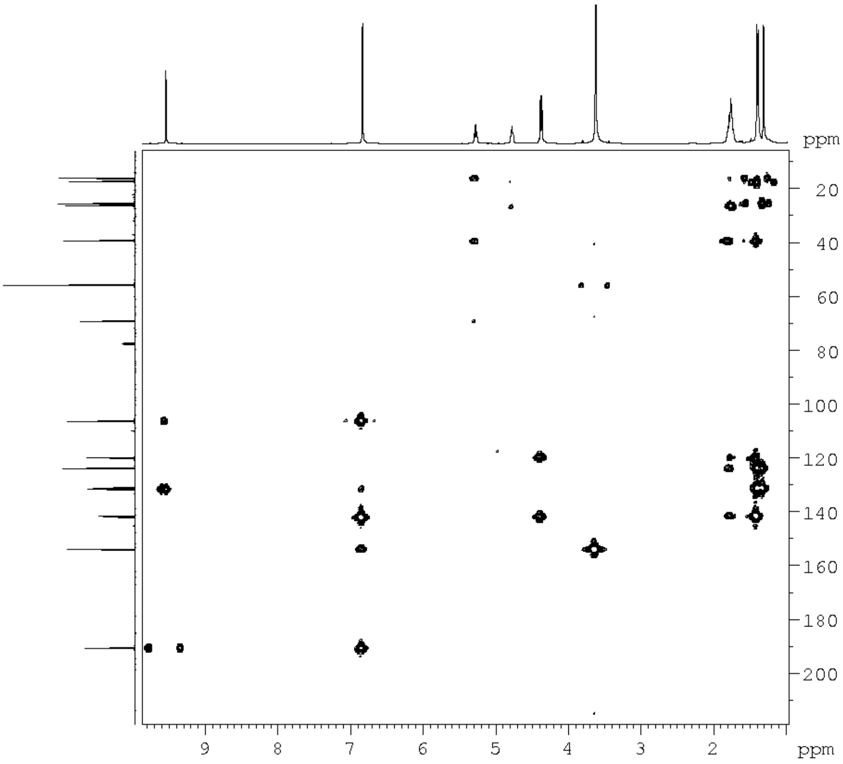

Figure S22. 2D HMBC spectrum compound **15**

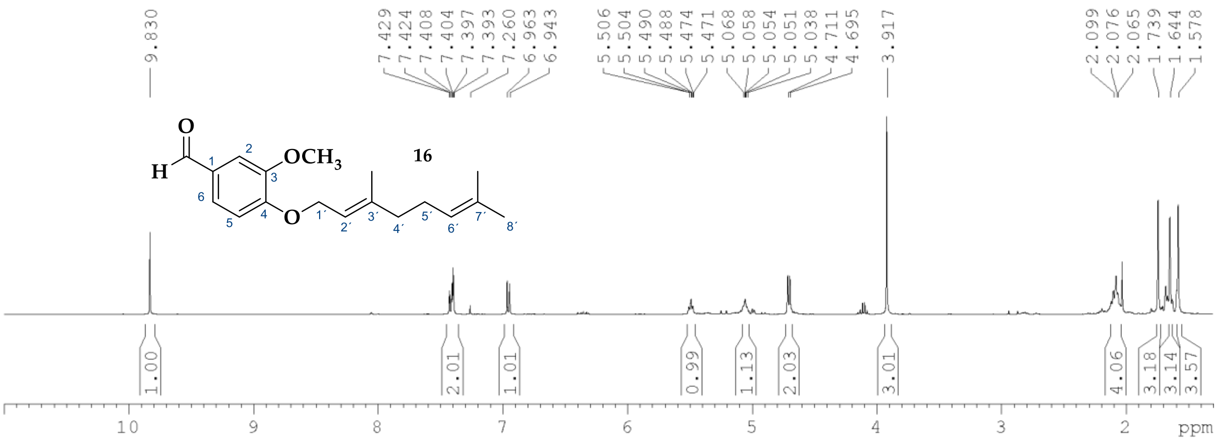

Figure S23. ^{1}H NMR spectrum compound **16**

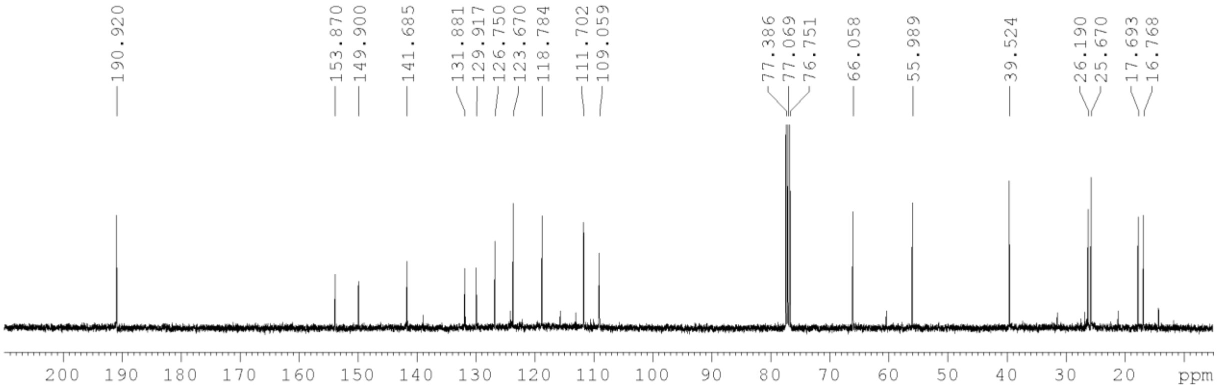

Figure S24. ^{13}C NMR spectrum compound **16**

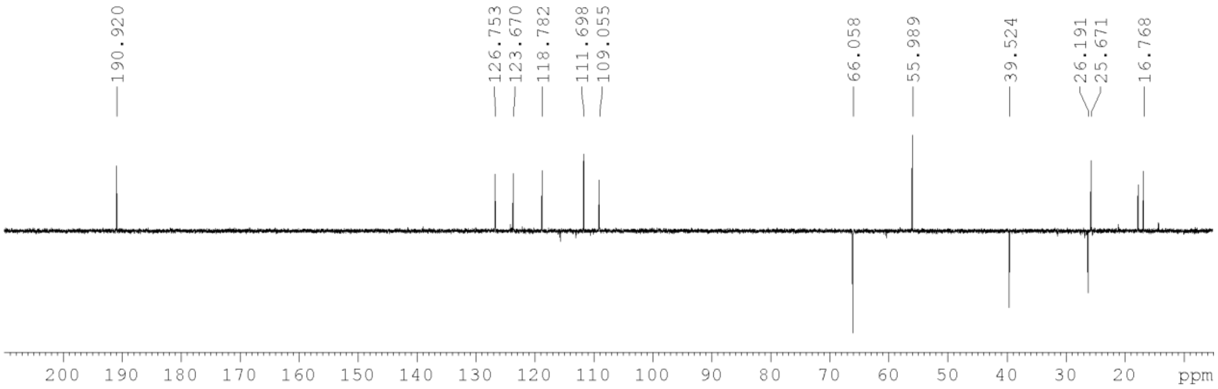

Figure S25. ^{13}C DEPT-135 NMR spectrum of **16**

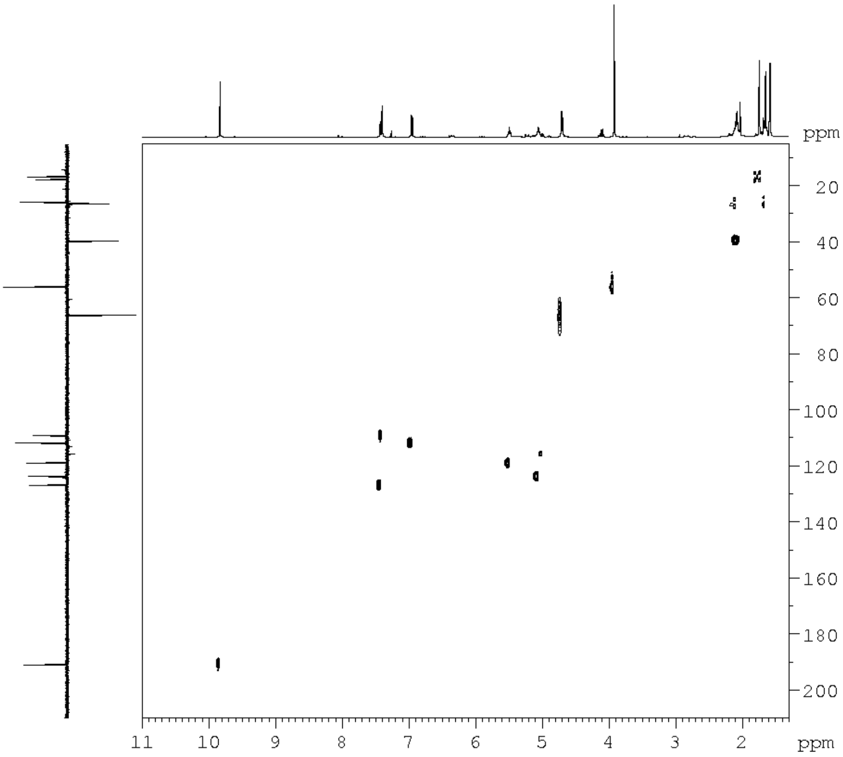

Figure S26. 2D HSQC NMR spectrum of **16**

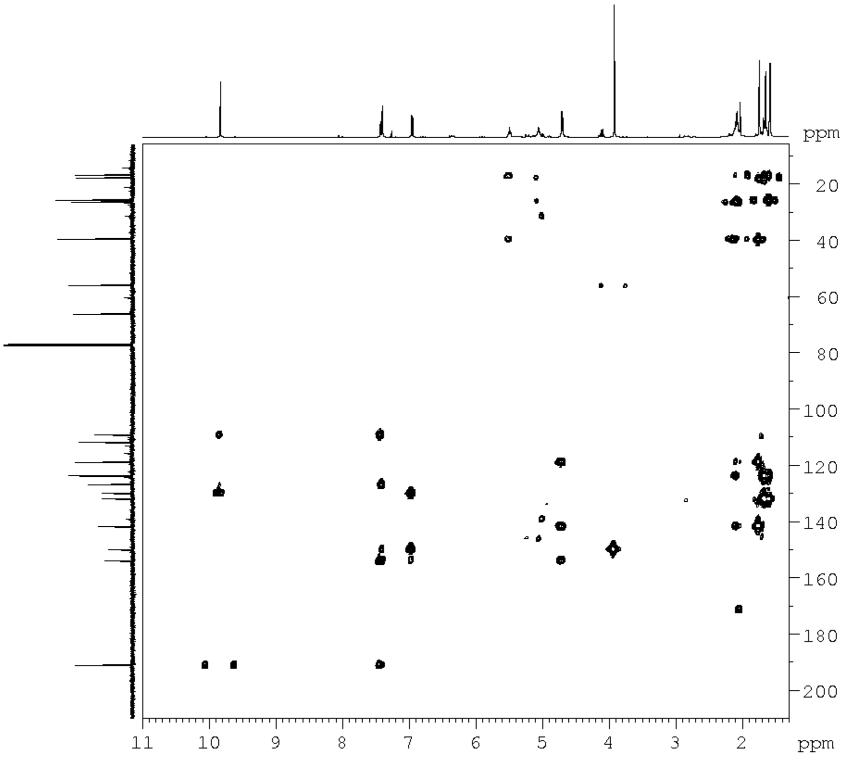

Figure S27. 2D HMBC spectrum compound **16**

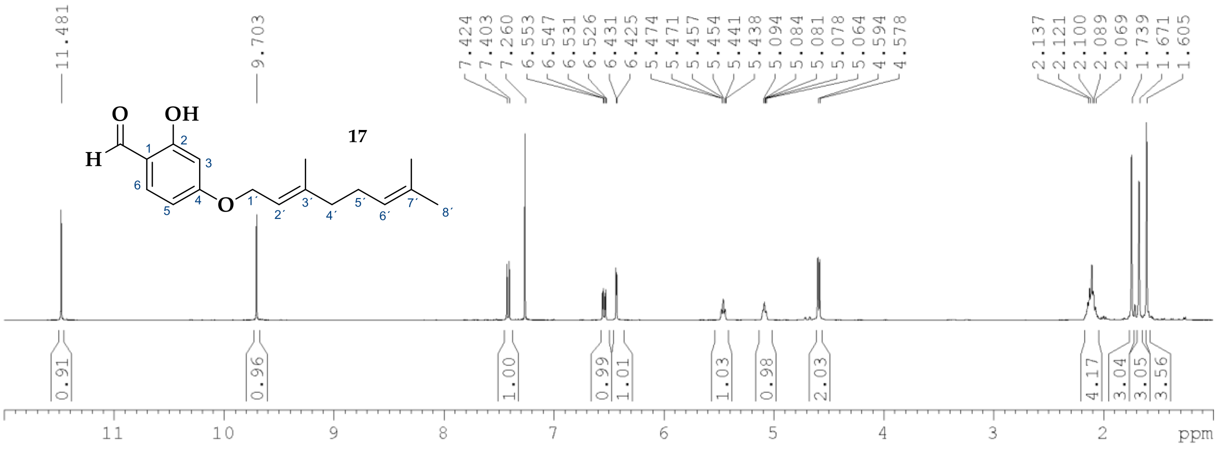

Figure S28. ^{1}H NMR spectrum compound **17**

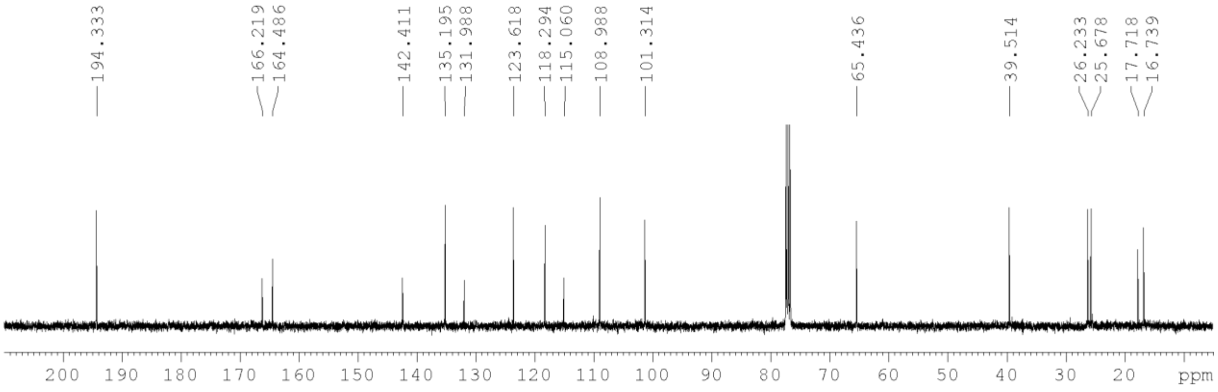

Figure S29. ^{13}C NMR spectrum compound **17**

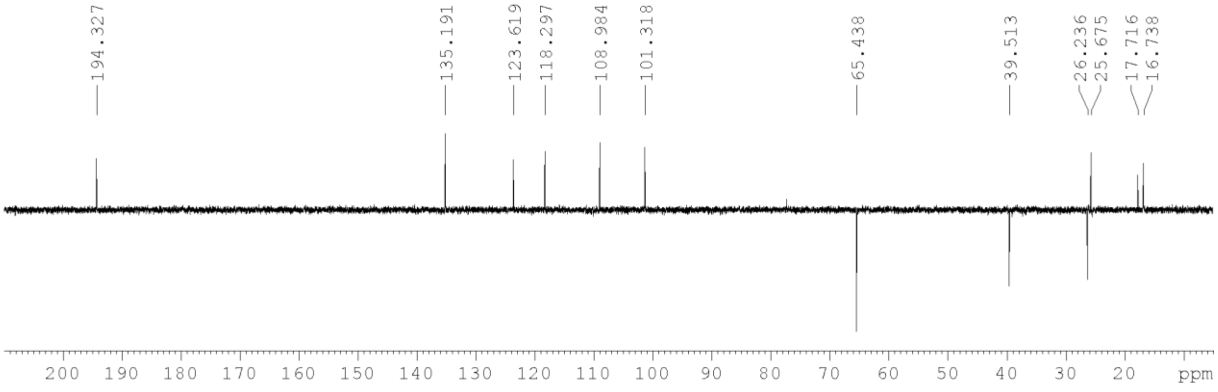

Figure S30. ^{13}C DEPT-135 NMR spectrum of **17**

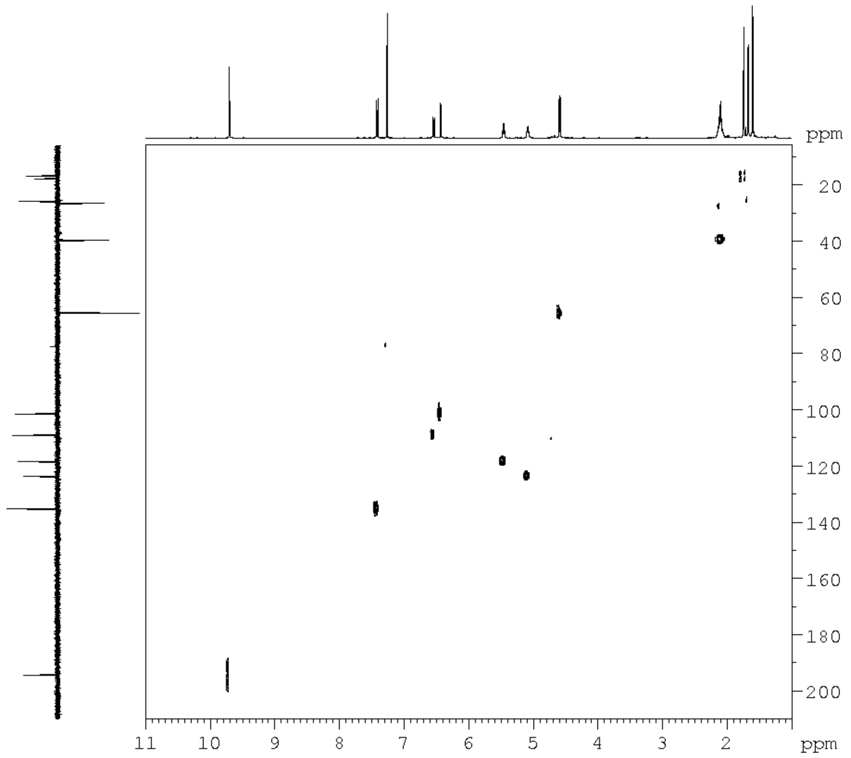

Figure S31. 2D HSQC NMR spectrum of **17**

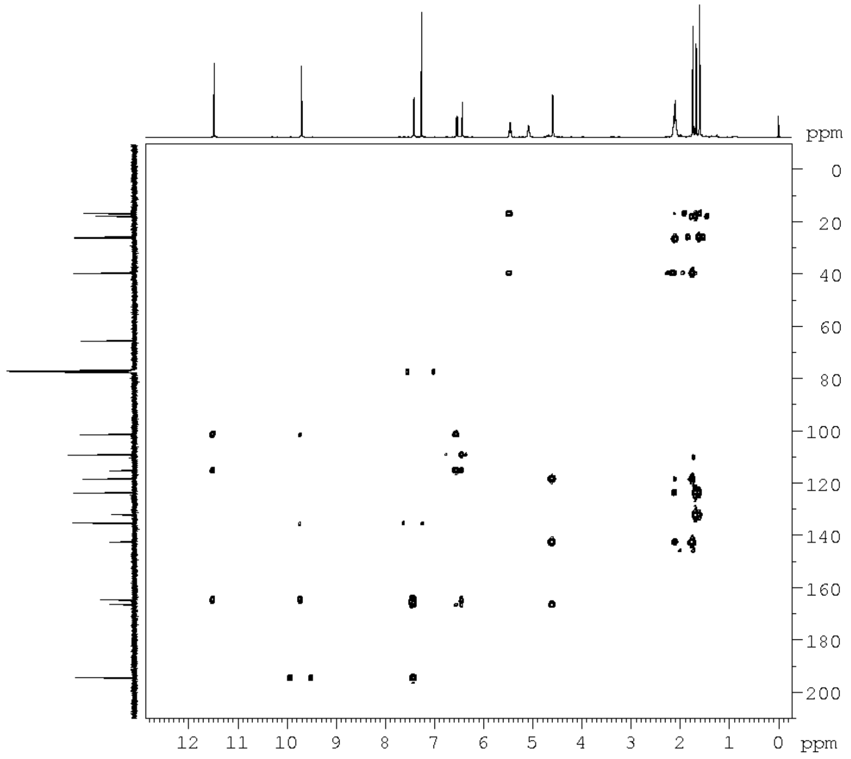

Figure S32. 2D HMBC spectrum compound **17**

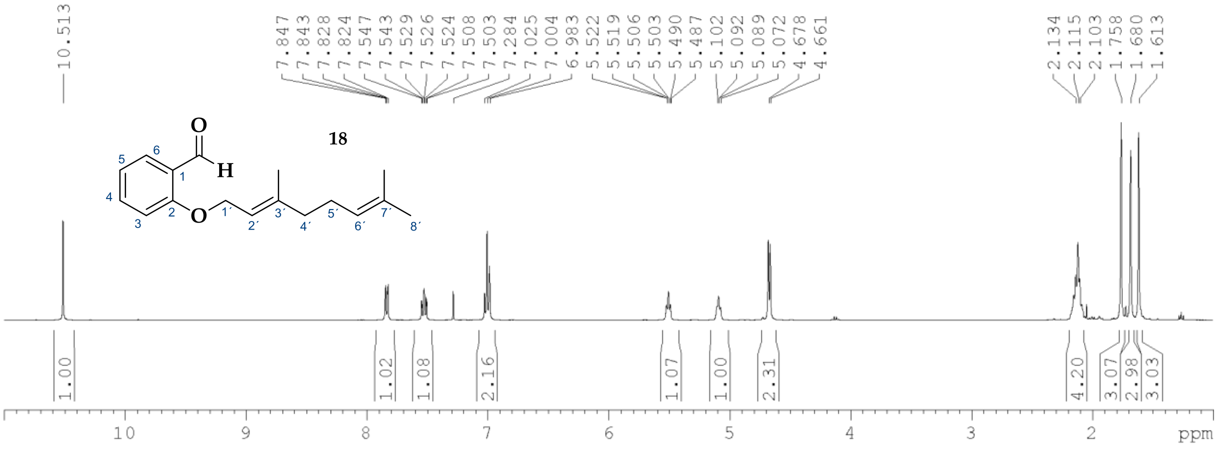

Figure S33. ^{1}H NMR spectrum compound **18**

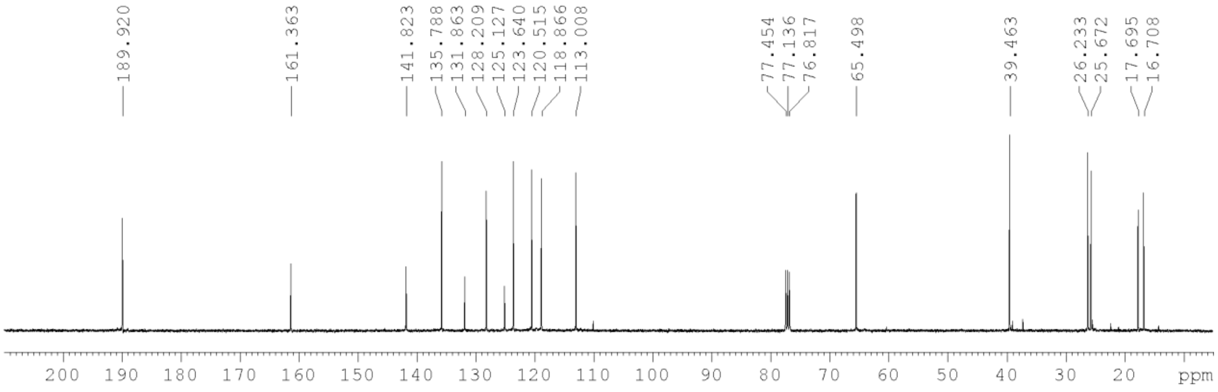

Figure S34. ^{13}C NMR spectrum compound **18**

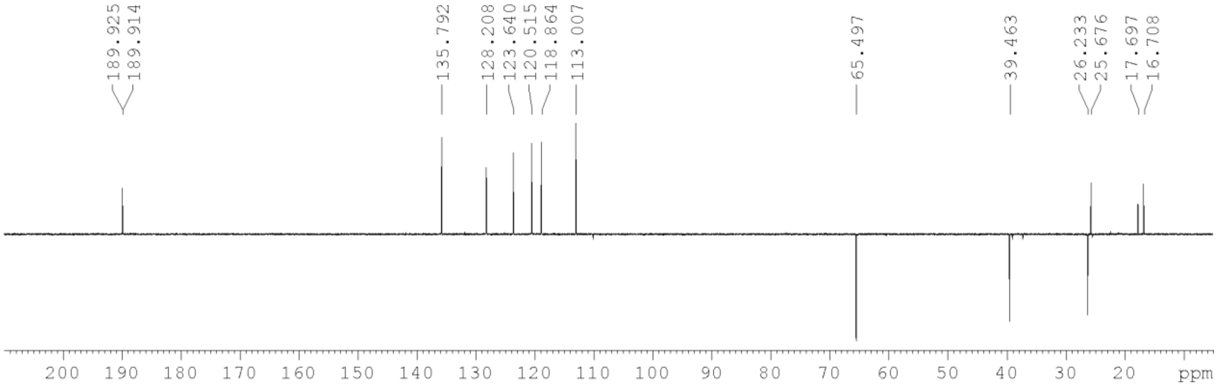

Figure S35. ^{13}C DEPT-135 NMR spectrum of **18**

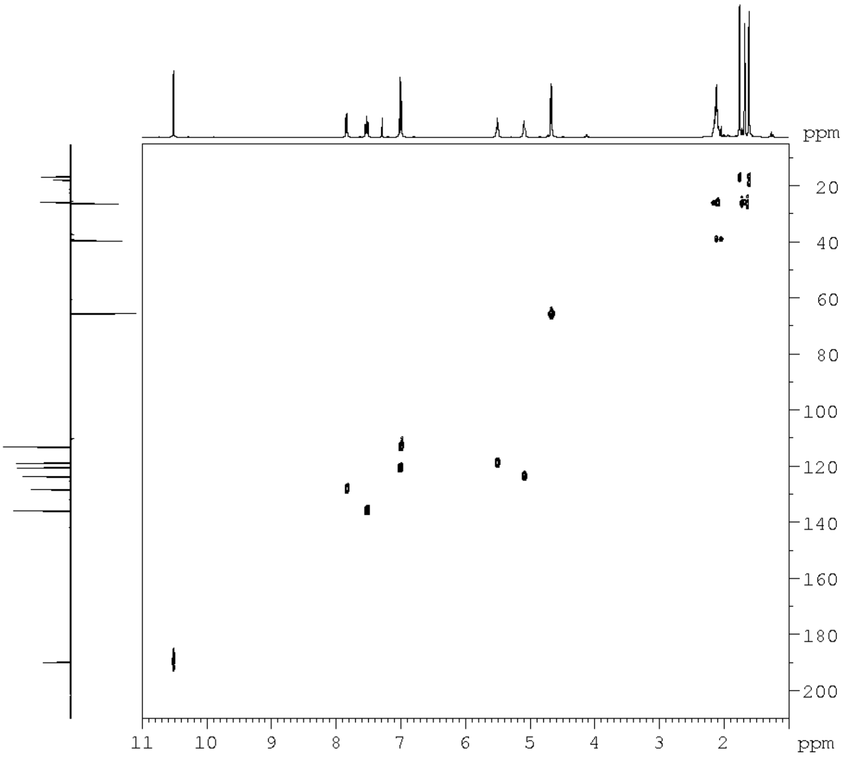

Figure S36. 2D HSQC NMR spectrum of **18**

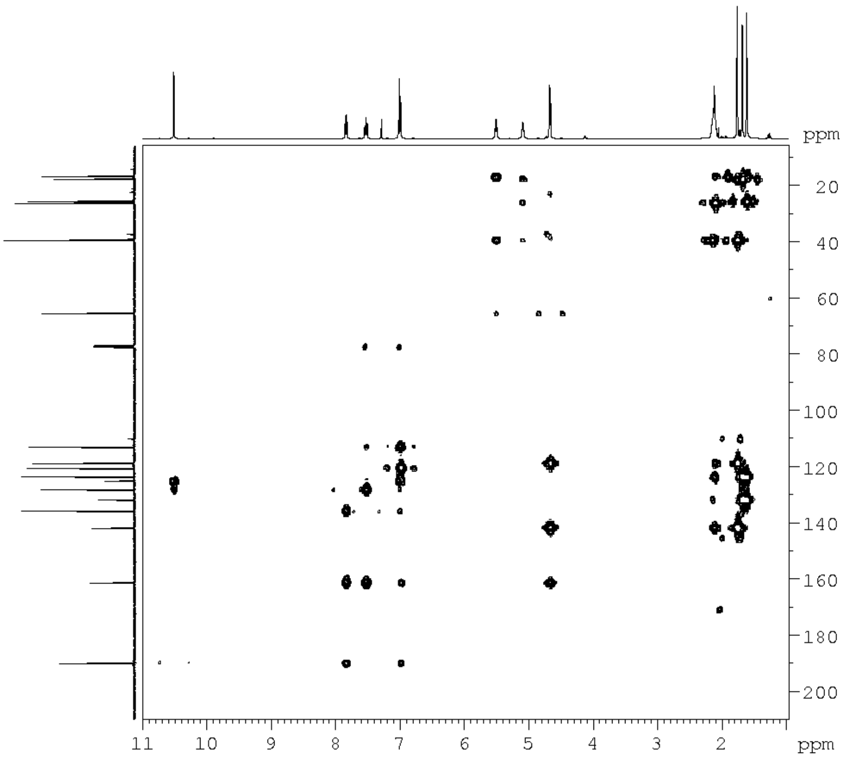

Figure S37. 2D HMBC spectrum compound **18**

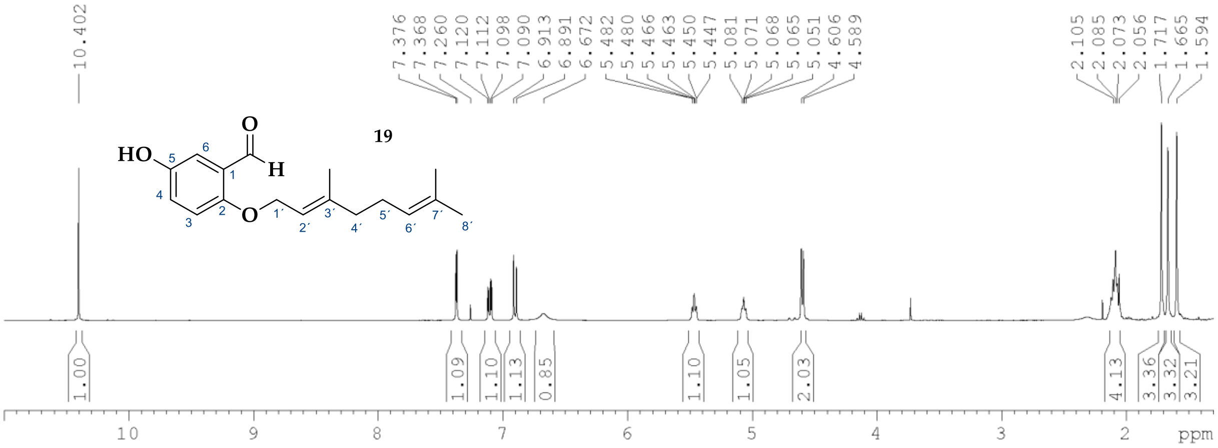

Figure S38. ^{1}H NMR spectrum compound **19**

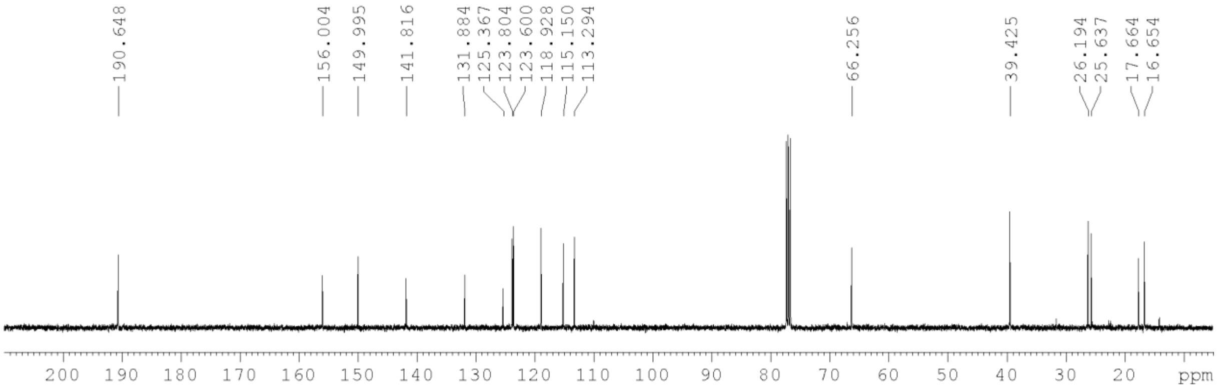

Figure S39. ^{13}C NMR spectrum compound **19**

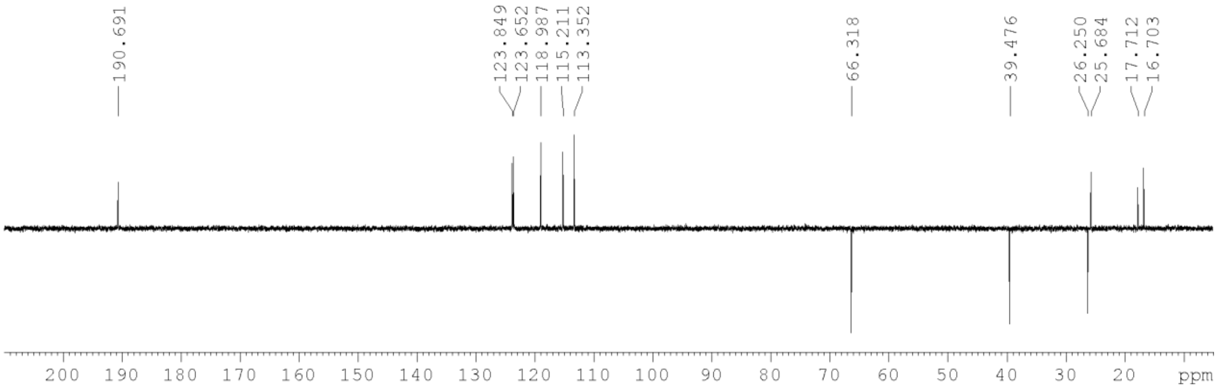

Figure S40. ^{13}C NMR spectrum compound **19**

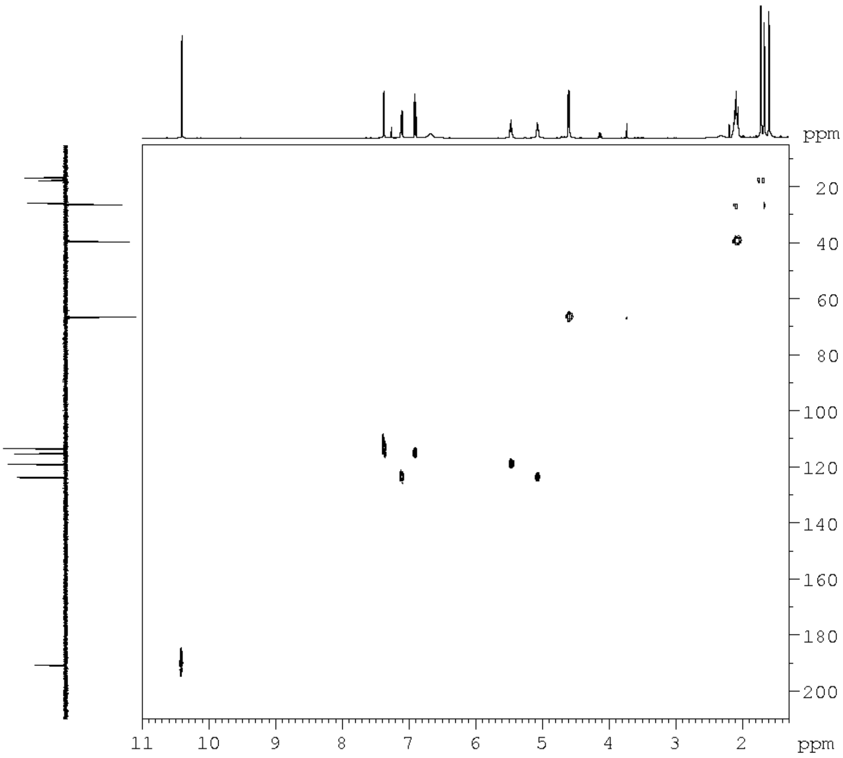

Figure S41. 2D HSQC NMR spectrum of **19**

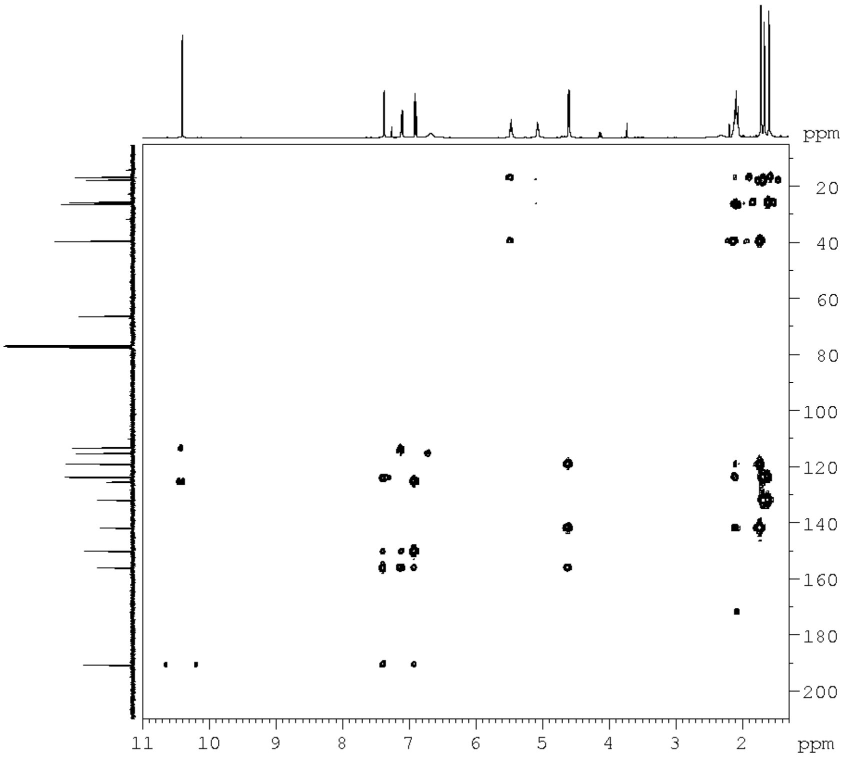

Figure S42. 2D HMBC spectrum compound **19**

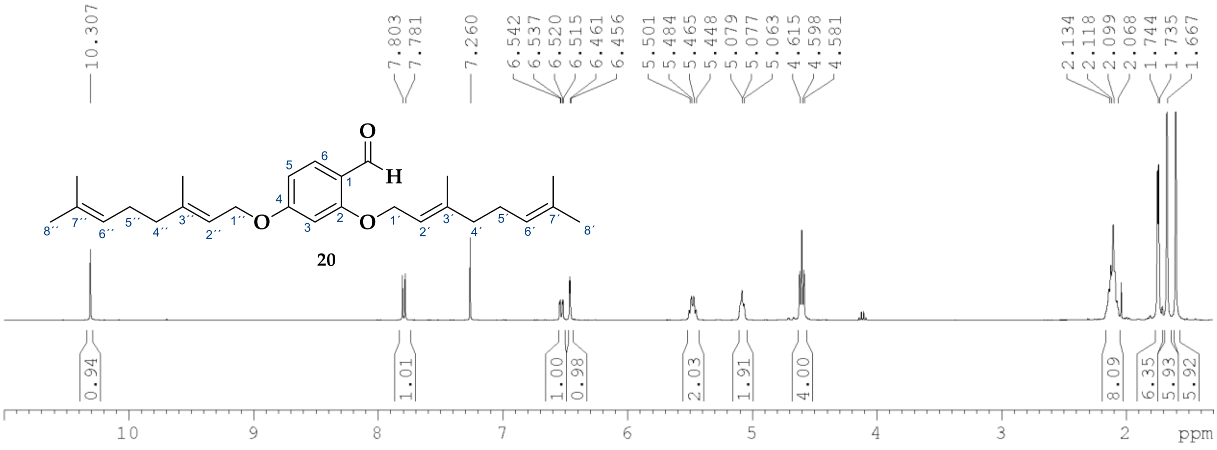

Figure S43. ^{1}H NMR spectrum compound **20**

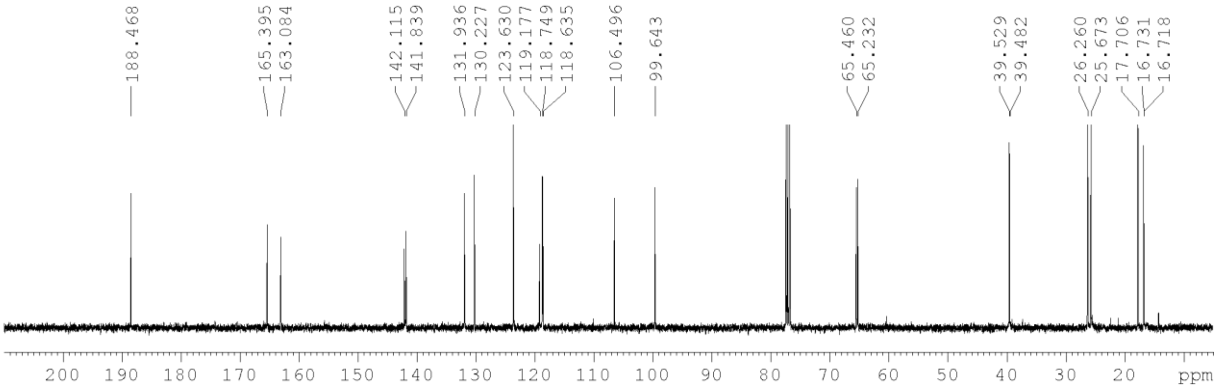

Figure S44. ^{13}C NMR spectrum compound **20**

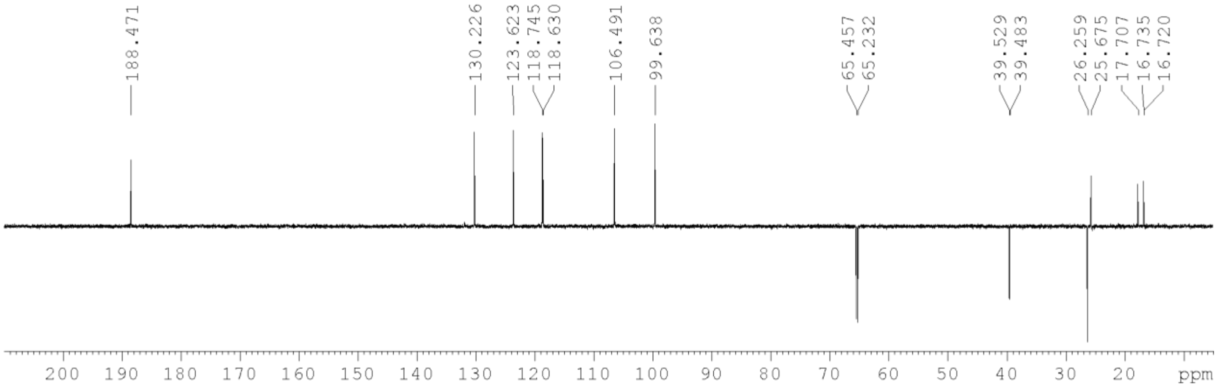

Figure S45. ^{13}C NMR spectrum compound **20**

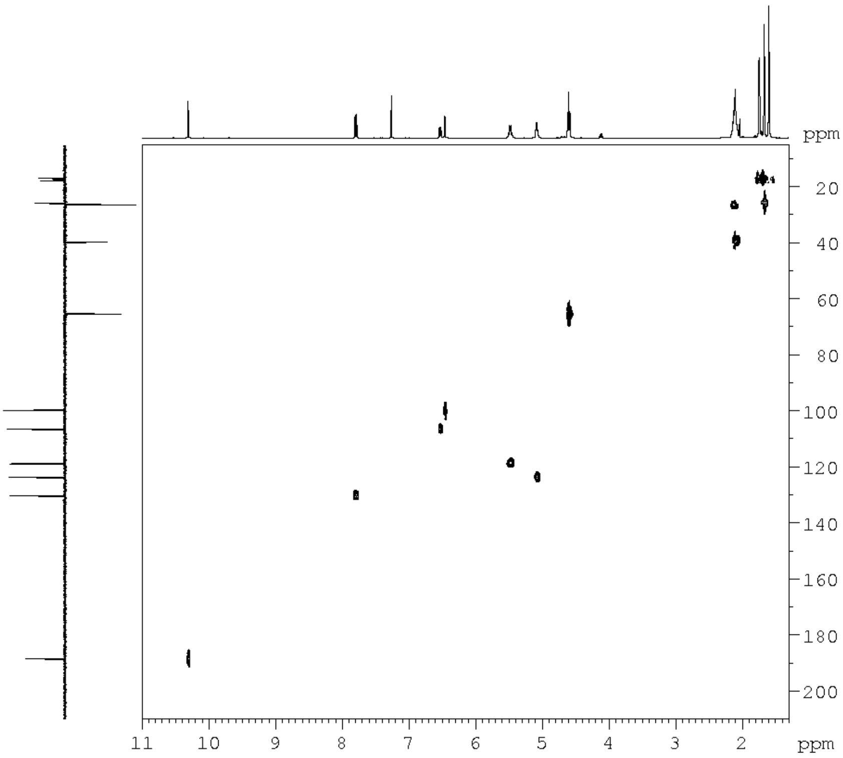

Figure S46. 2D HSQC NMR spectrum of **20**

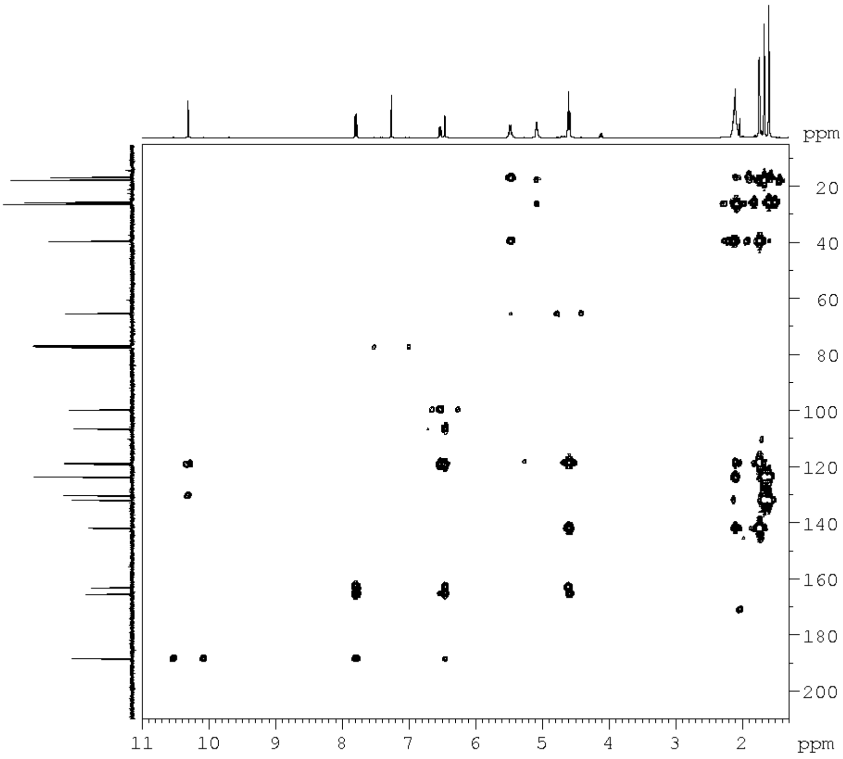

Figure S47. 2D HMBC spectrum compound **20**

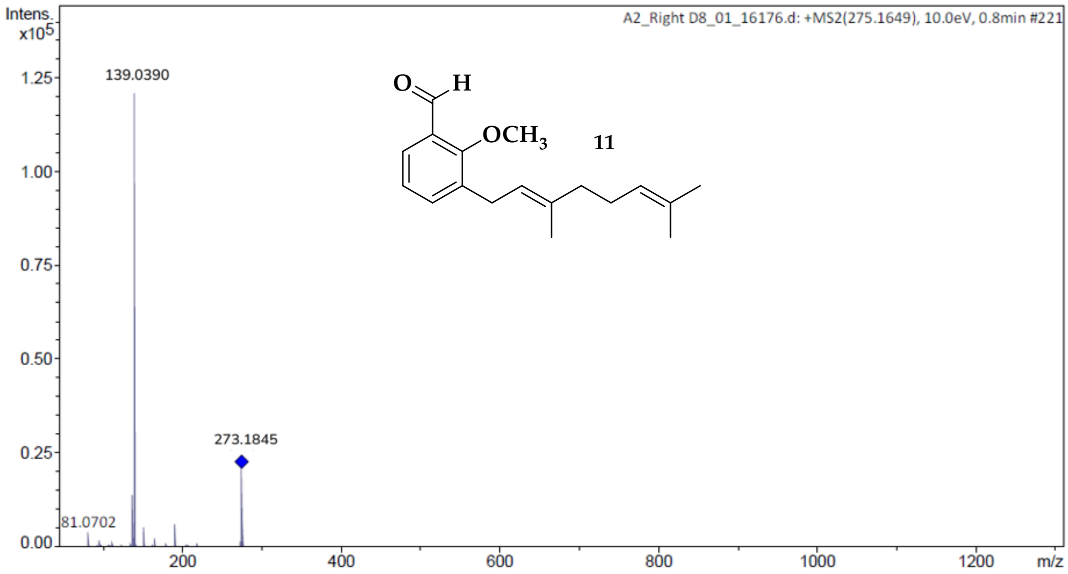

Figure S48. HRSM spectrum of compound **11**

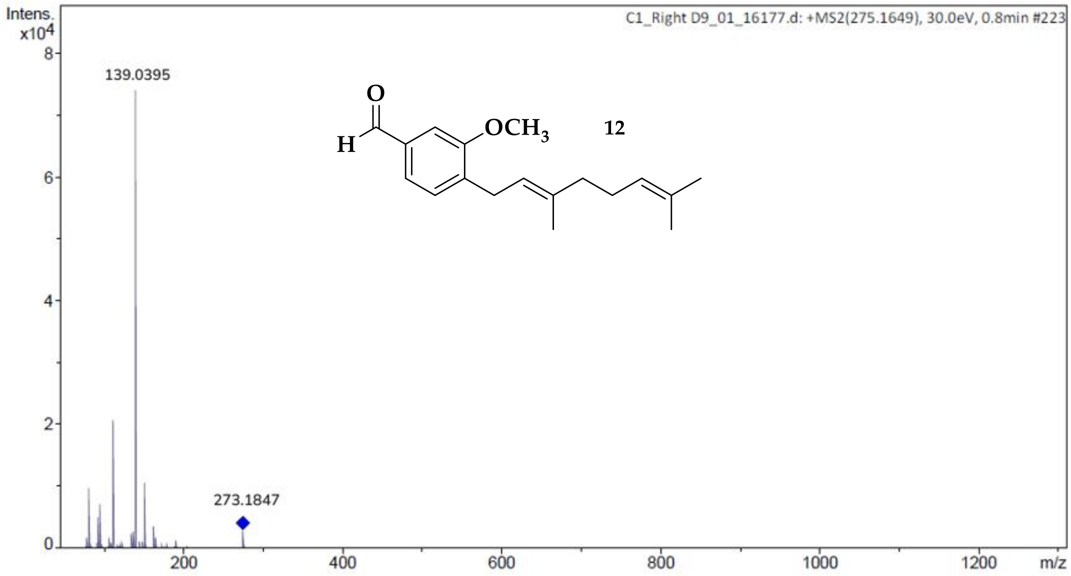

Figure S49. HRSM spectrum of compound **12**

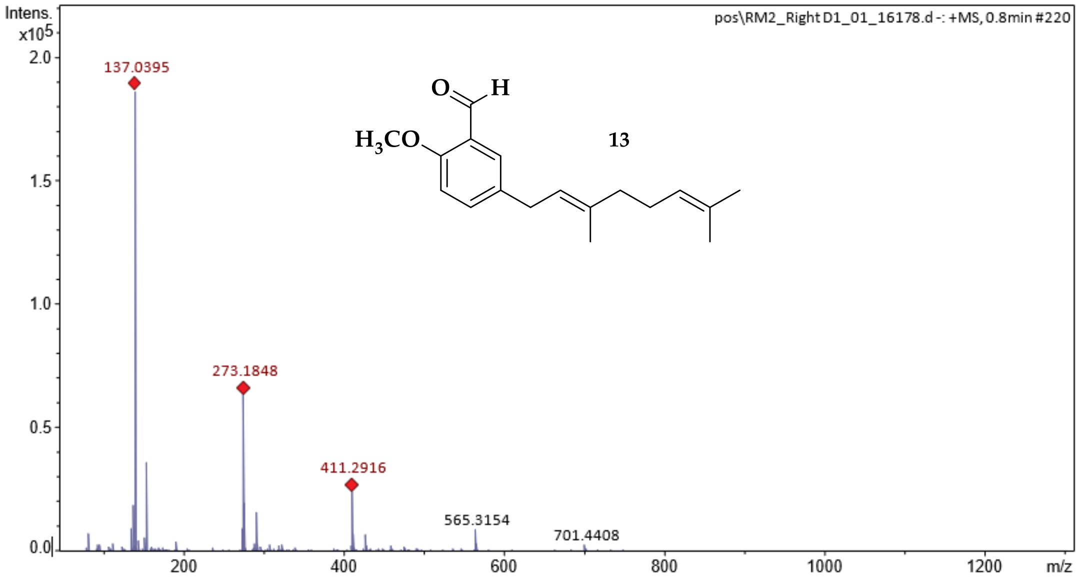

Figure S50. HRSM spectrum of compound **13**

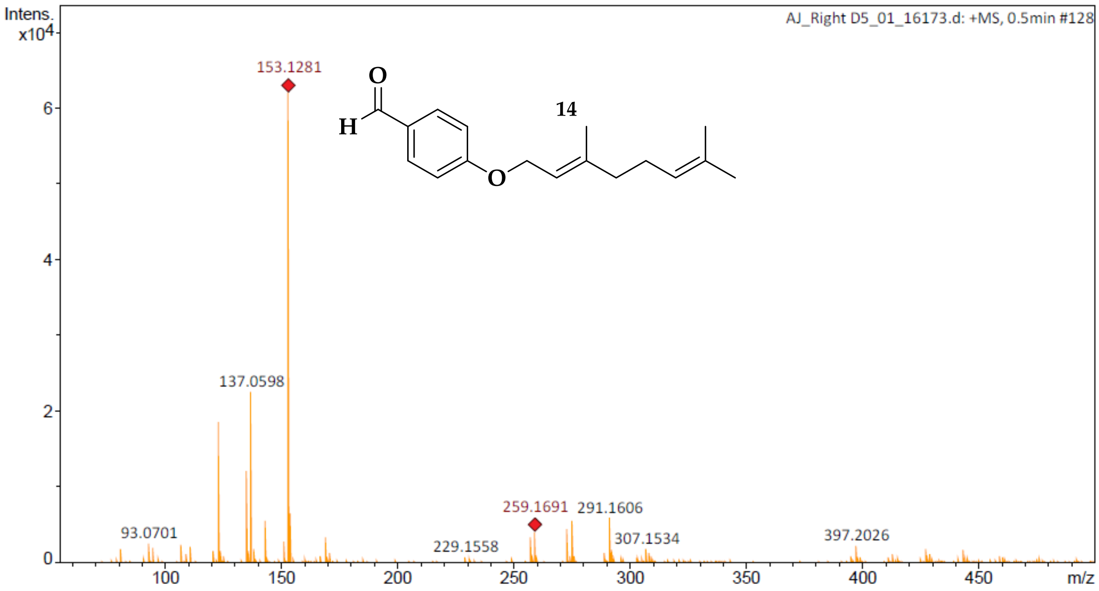

Figure S51. HRSM spectrum of compound **14**

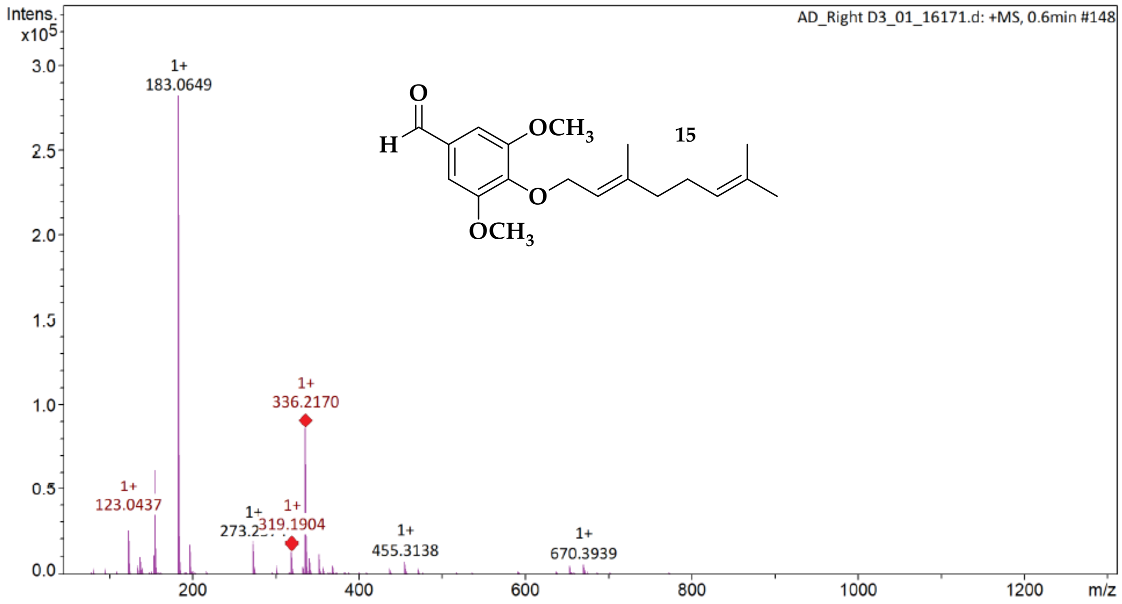

Figure S52. HRSM spectrum of compound **15**

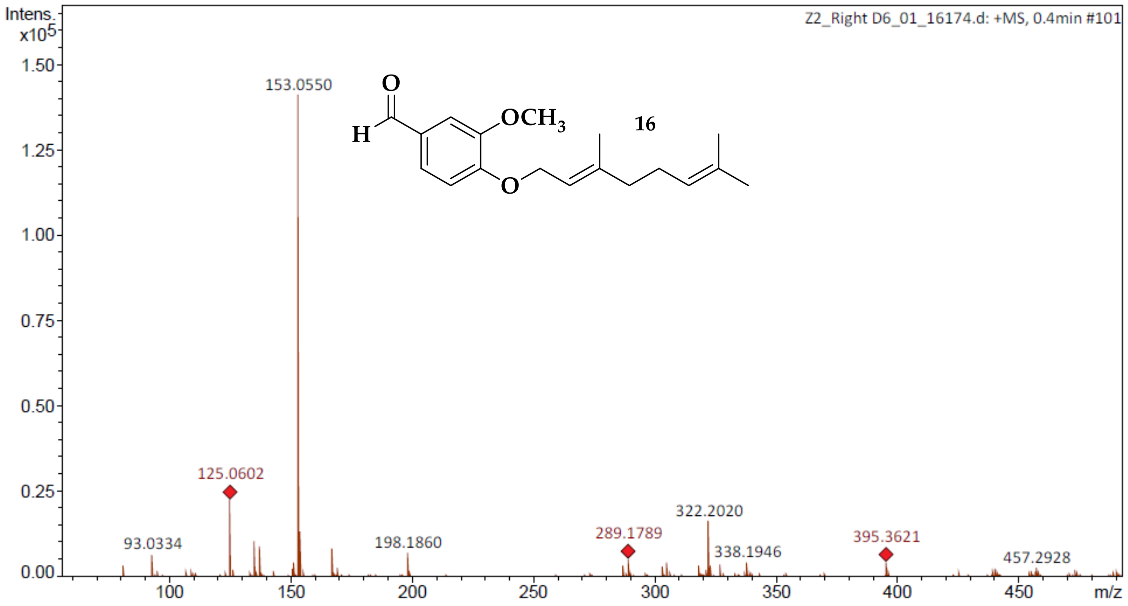

Figure S53. HRSM spectrum of compound **16**

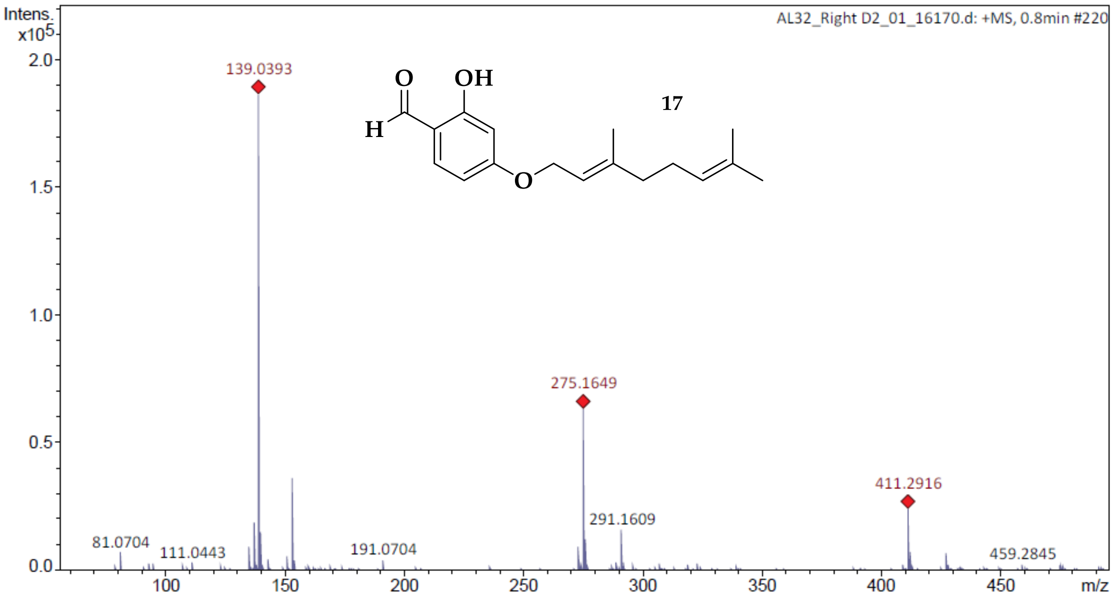

Figure S54. HRSM spectrum of compound **17**

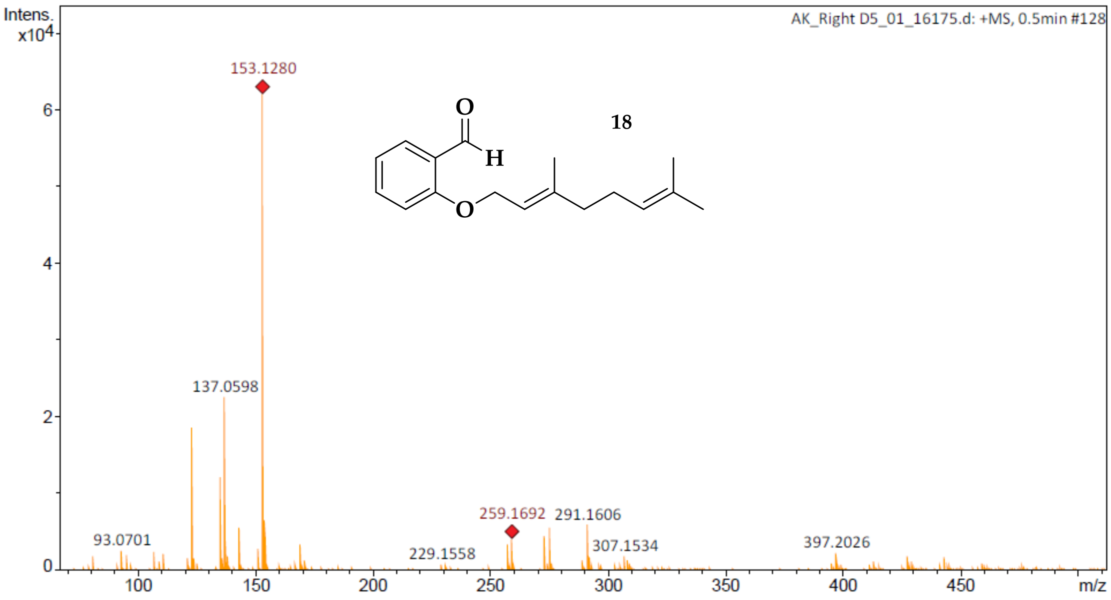

Figure S55. HRSM spectrum of compound **18**

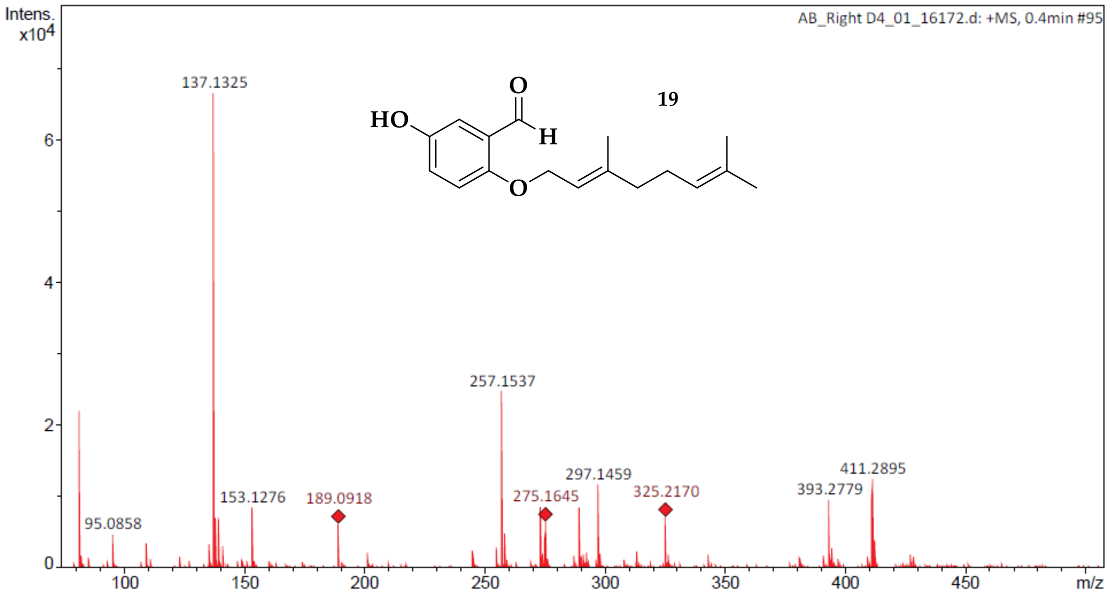

Figure S56. HRSM spectrum of compound **19**

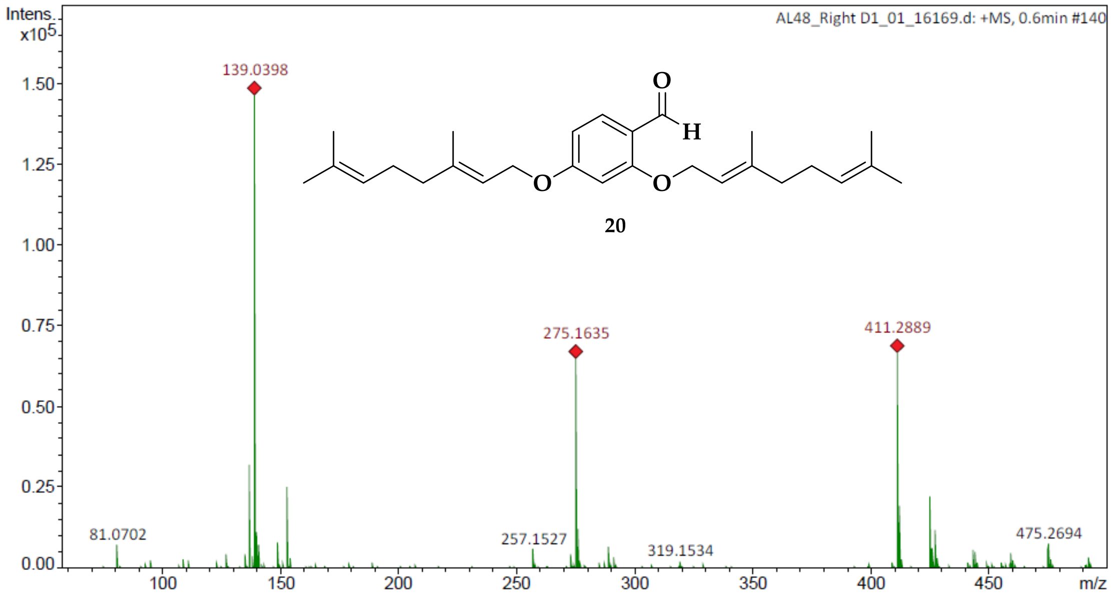

Figure S57. HRSM spectrum of compound **20**

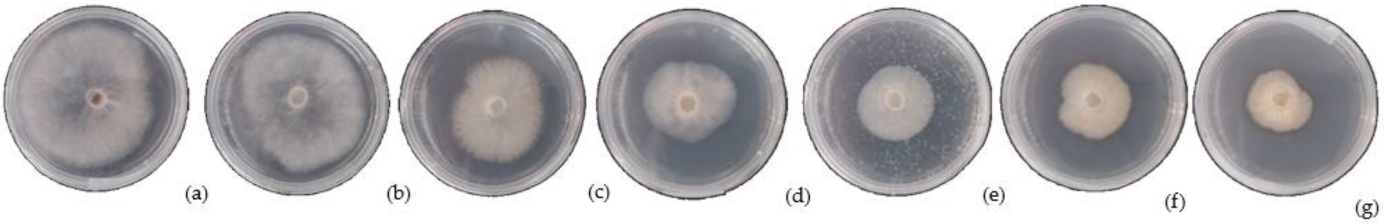

Figure S58. In vitro effect of geranylated benzaldehyde (**19**) on mycelial growth of *B. cinerea* at different concentrations: (a) 0 µg/mL (Negative control); (b) solvent; (c) 10 µg/mL; (d) 20 µg/mL; (e) 40 µg/mL; (f) 80 µg/mL; (g) 160 µg/mL.

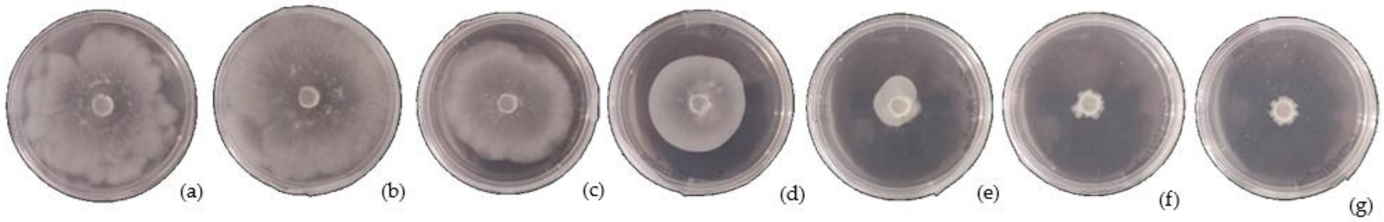

Figure S59. *In vitro* effect of geranylated benzaldehyde (**19**) on mycelial growth of *P. cinnamomi* at different concentrations: (PAa) 0 µg/mL (Negative control); (b) solvent; (c) 3 µg/mL; (d) 6 µg/mL; (e) 12 µg/mL; (f) 25 µg/mL; (g) 50 µg/mL.

For all molecular docking images, the following representations were used: hydrogen bonds are depicted as green dashed lines; π–π stacking interactions are depicted as dark pink dashed lines; and hydrophobic interactions are depicted as pink dashed lines. Visualization of the docked poses was performed using Discovery Studio Visualizer (BIOVIA, San Diego, CA, USA).

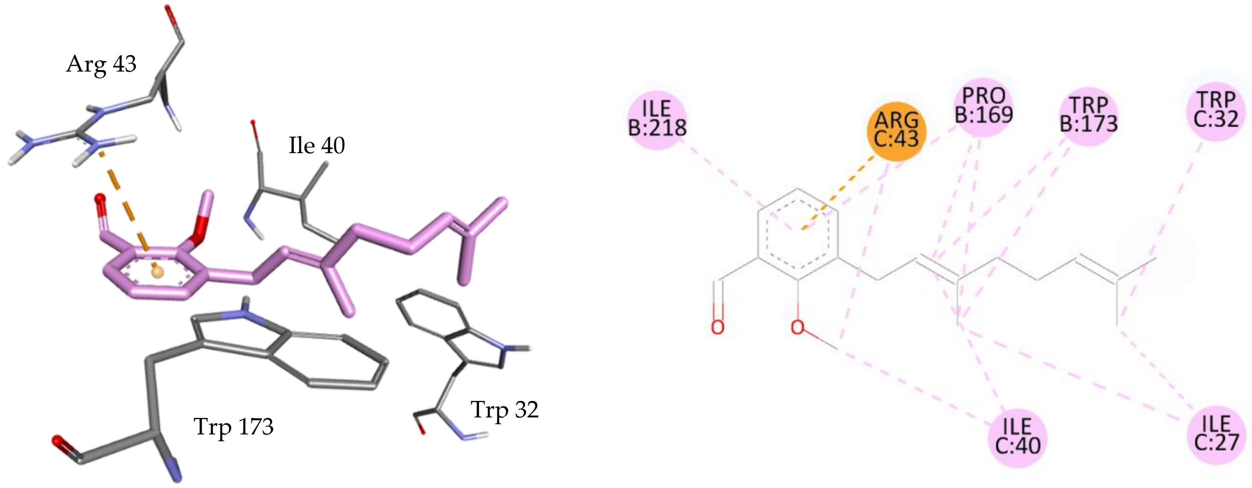

Figure S60. Protein-ligand interactions with Compound **11**

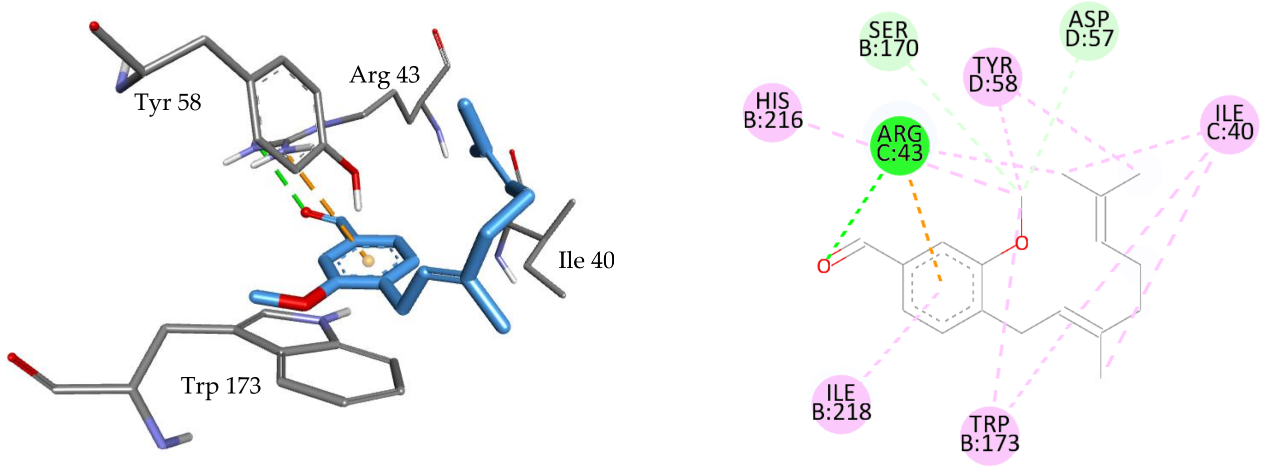

Figure S61. Protein-ligand interactions with Compound **12**

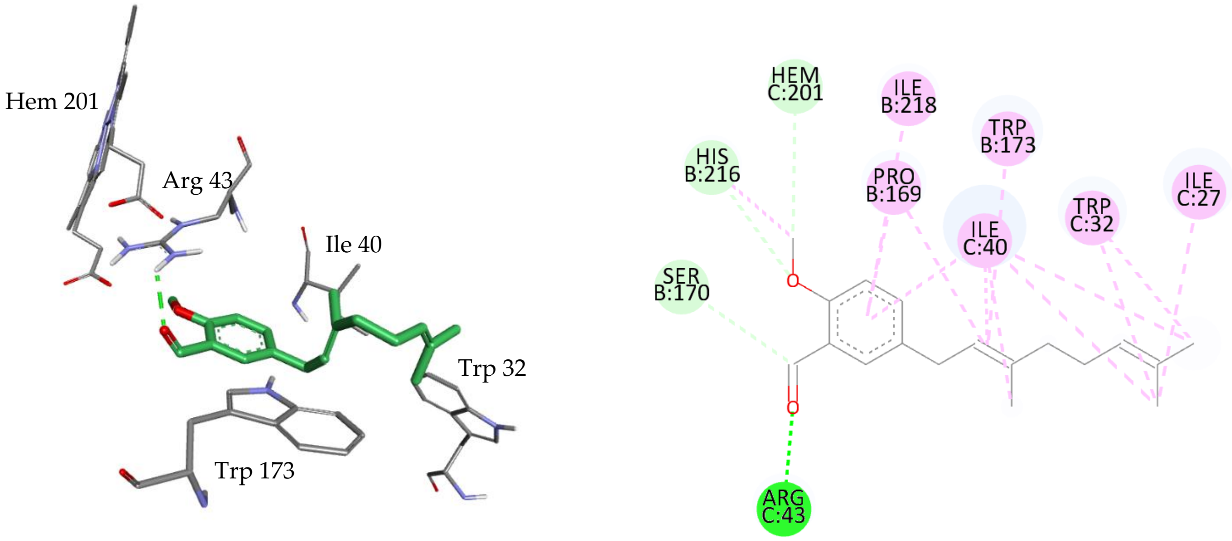

Figure S62. Protein-ligand interactions with Compound **13**

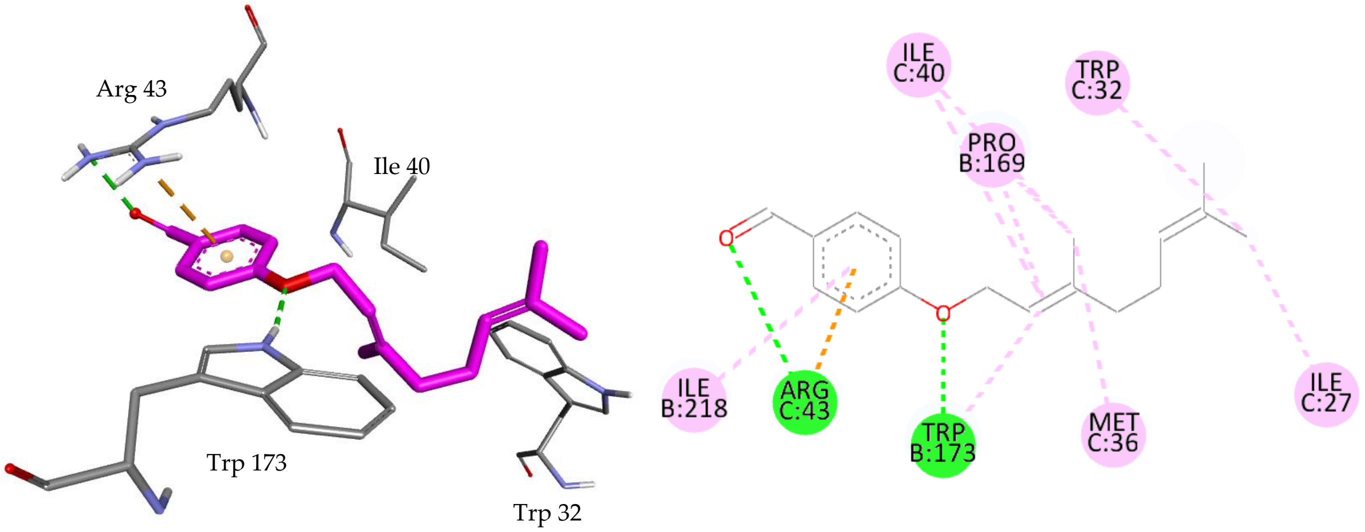

Figure S63. Protein-ligand interactions with Compound **14**

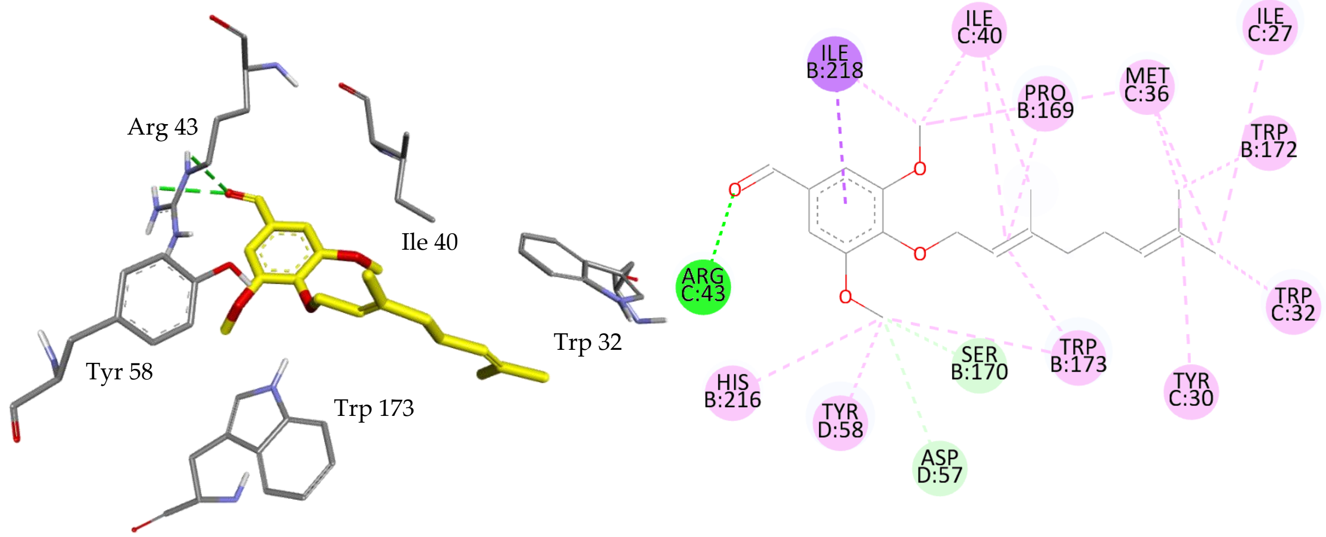

Figure S64. Protein-ligand interactions with Compound **15**

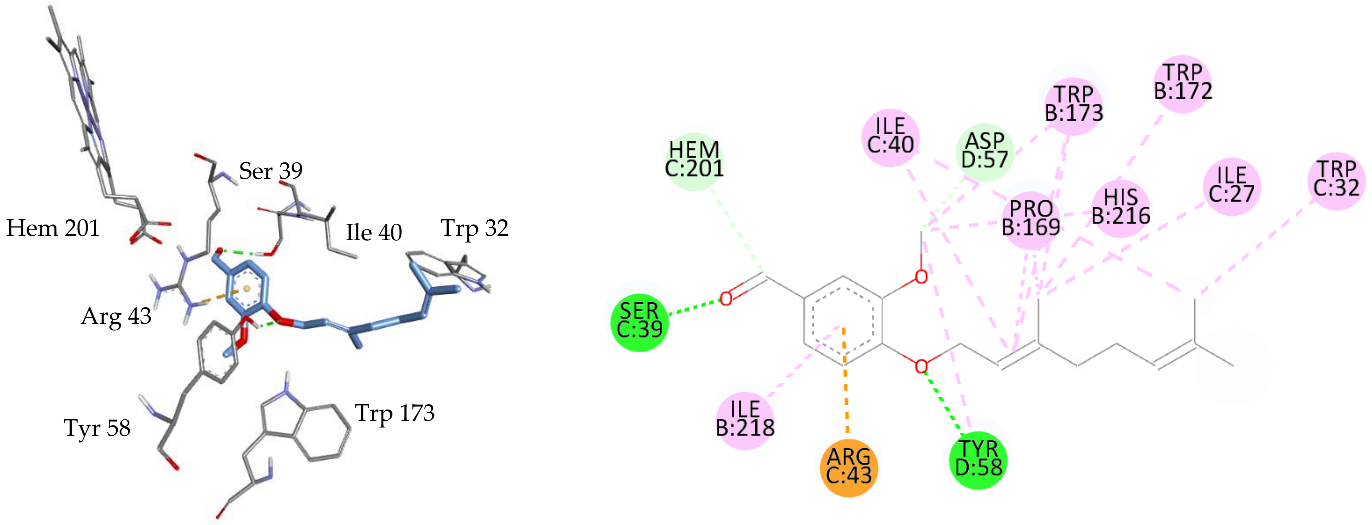

Figure S65. Protein-ligand interactions with Compound **16**

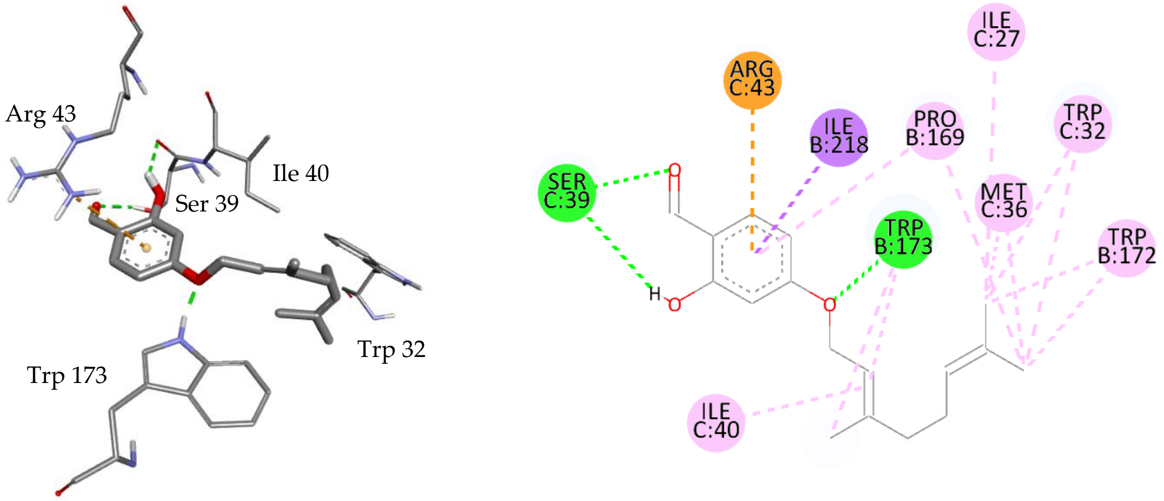

Figure S66. Protein-ligand interactions with Compound **17**

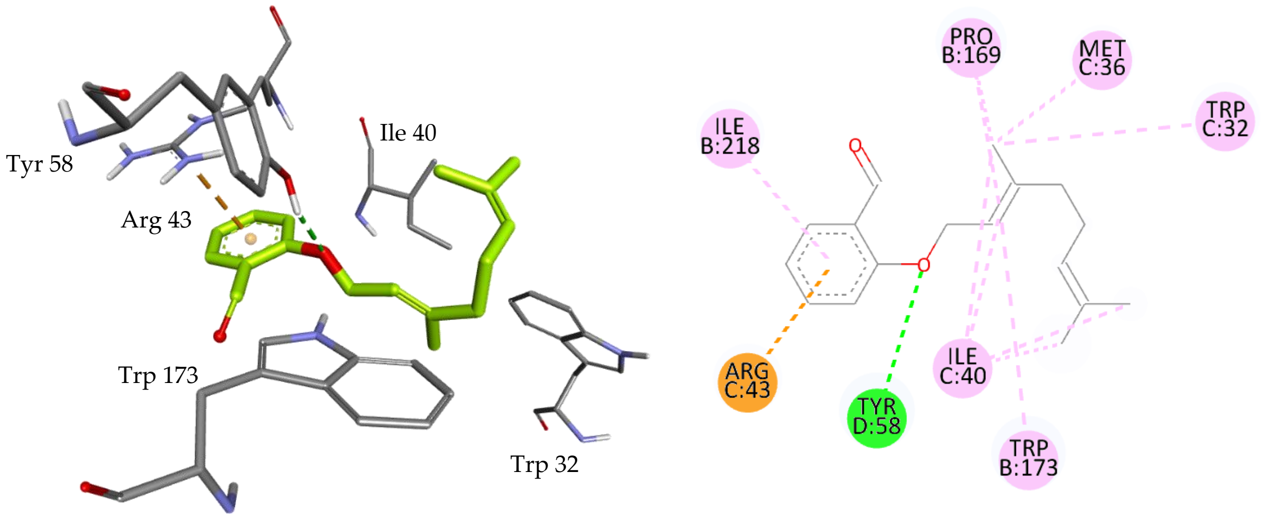

Figure S67. Protein-ligand interactions with Compound **18**

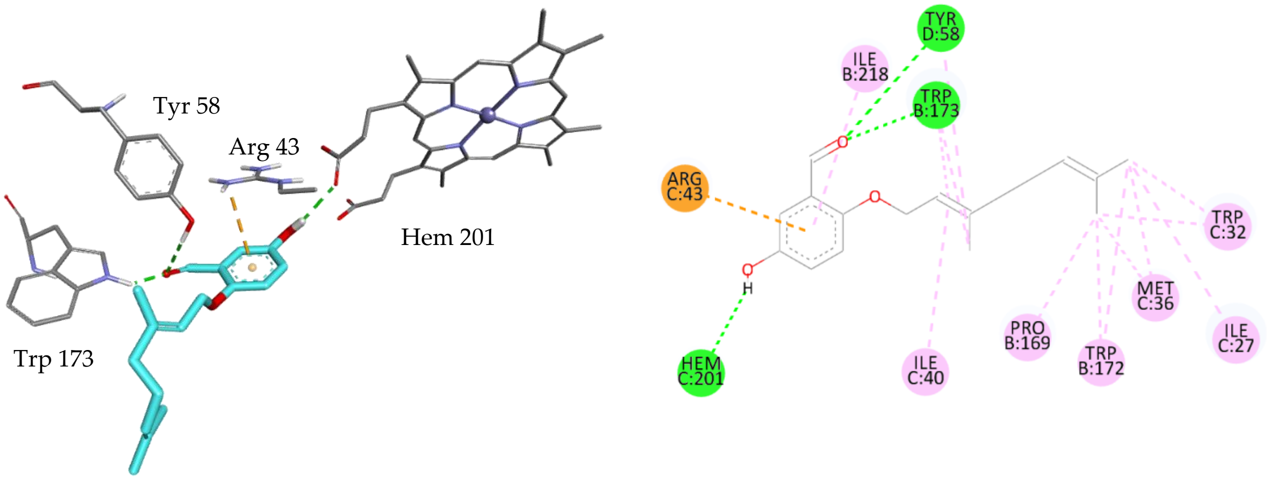

Figure S68. Protein-ligand interactions with Compound **19**

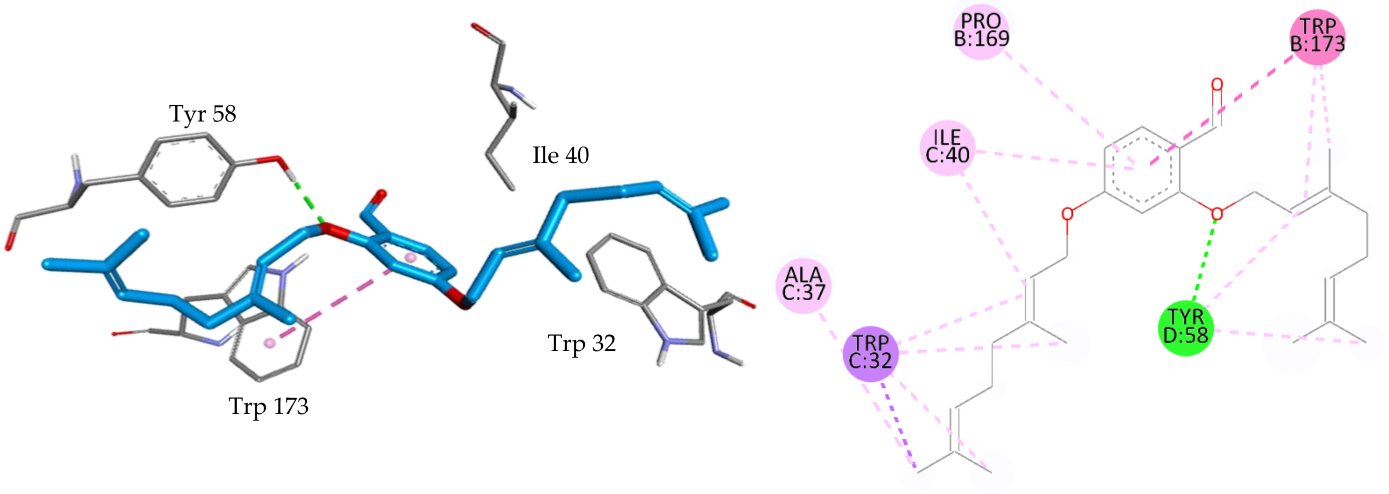

Figure S69. Protein-ligand interactions with Compound **20**

Table S1. Molecular interactions of compounds **9–18** within the SDH active site.

Compound	Protein Contacts	
	Hydrogen bonds	Non-polar interactions
**11**	-	Ile218, Pro169, Trp173, Trp32, Ile40, Ile27, Arg43
**12**	Arg43	His216, Tyr58, Ile40, Trp173, Ile218
**13**	Arg43	His216, Ile218, Pro169, Trp173, Trp32, Ile27, Ile40
**14**	Arg43<br>Trp173	Ile218, Trp173, Met36, Pro169, Ile40, Trp32, Ile27
**15**	Arg43	His216, Tyr58, Trp173, Tyr30, Trp32, Trp172, Ile27, Met36, Pro169, Ile40, Ile218
**16**	Ser39<br>Tyr58	Ile218, Ile40, Pro169, Trp173, His216, Ile27, Trp32, Trp172, Tyr58
**17**	Ser39<br>Ser39<br>Trp173	Ile40, Trp172, Trp32, Met36, Ile27, Pro169, Ile218, Trp173, Arg43
**18**	Tyr58	Ile218, Pro169, Met36, Trp32, Ile40, Trp173, Arg43
**19**	Tyr58<br>Trp173<br>Hem201	Arg43, Ile218, Tyr58, Trp173, Ile40, Pro169, Trp172, Met36, Ile27, Trp32
**20**	Tyr58	Ile40, Pro169, Trp173, Tyr58, Trp32, Ala37

Table S2. Pose analysis of docked synthetic analogs (**11**-**20**). ΔE_b: Binding Energy in kcal/mol.

**Code**	**ΔE_b**	**Structure**
**11**	-6.5	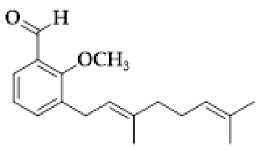
**12**	-6.8	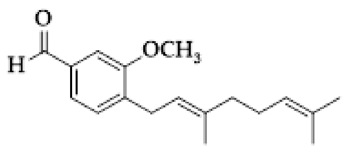
**13**	-6.8	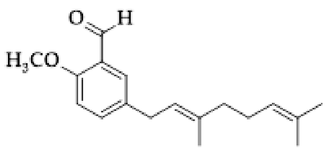
**14**	-6.8	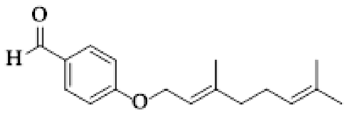

**15**	-6.8	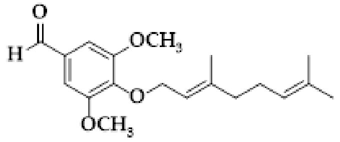
**16**	-6.9	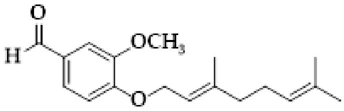
**17**	-7.0	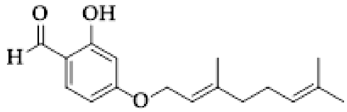
**18**	-6.8	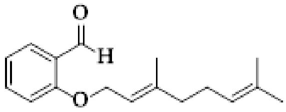
**19**	-7.5	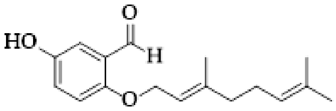
**20**	-6.0	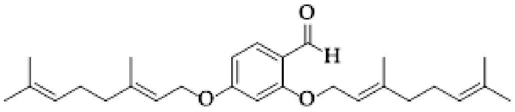
